# Clinical and cognitive assessment in Friedreich ataxia clinical trials: a review

**DOI:** 10.3389/fneur.2025.1558493

**Published:** 2025-05-22

**Authors:** Álvaro Darriba, Arnold Munnich, Pedro Cardoso-Leite, Benoît Funalot, Florian Waszak

**Affiliations:** ^1^Université Paris Cité, CNRS, UMR 8002 INCC, Paris, France; ^2^Institut Imagine, UMR 1163, Hôpital Necker and Université Paris Cité, Paris, France; ^3^Department of Behavioural and Cognitive Sciences, University of Luxembourg, Esch-sur-Alzette, Luxembourg; ^4^Department of Medical Genetics, Assistance Publique-Hôpitaux de Paris, Hôpital Necker Enfants Malades, Paris, France; ^5^Department of Medical Genetics, Assistance Publique-Hôpitaux de Paris, Henri Mondor University Hospital, Créteil, France; ^6^IMRB, INSERM U955, Université Paris-Est Créteil, Creteil, France

**Keywords:** Friedreich’s ataxia, behavioral assessment, cognitive assessment, clinical trials, outcome measures

## Abstract

Friedreich ataxia (FRDA) is the most common type of inherited ataxia. It is a neurodegenerative disorder characterized by progressive gait and limb ataxia, dysarthria, areflexia, and reduced proprioception and vibration sensation. Although a number of clinical trials have been conducted, there is currently no cure for this disease. In this article we review those clinical trials with a focus on the instruments used as endpoints to assess clinical progression, and discuss the potential benefits of integrating additional measures, including assessments from overlooked domains. We also review tools used to evaluate cognitive functions in individuals with FRDA, particularly those employing quantitative, objective, and time-based measures. We argue for the inclusion of cognitive and speech-related assessments in clinical trials, and examine the potential of developments in cognitive neuroscience and technology to address current measurement challenges and support more accurate and comprehensive evaluation of treatment effects. These innovations have the potential to complement existing approaches, enhance trial design, and advance clinical care.

## 1 Introduction

Friedreich’s ataxia (FRDA) is an autosomal recessive ataxia, typically characterized by gait and limb ataxia, dysarthria, loss of lower limb reflexes, extensor plantar responses, and reduced vibration sense and proprioception (1). It is the most common of inherited ataxias, and is a progressive multisystem disorder that impacts the central and peripheral nervous systems, musculoskeletal system, myocardium, and pancreas, causing multiple signs and symptoms (2). Neurological manifestations are linked to peripheral nervous system involvement and cerebellar degeneration, particularly of the dentate nuclei (3), which connect the cerebellum to a wide array of cortical areas (4). These cortico-cerebellar loops are essential for motor control and cognitive processes (5). Symptoms typically emerge when cerebellar signs appear (6), and worsen as the disease progresses (7).

The severity and progression of FRDA are commonly assessed using clinical rating scales (8). These scales primarily evaluate motor dysfunction, reflecting the traditional view of the cerebellum as a fundamentally motor control centre (9) and the confounding effects of motor impairments on assessing other functions, such as cognitive or affective domains (10). Of these scales, only one includes non- physical components, such as activities of daily living (11). While these tools demonstrate good validity and test–retest reliability (12–15), their sensitivity to detect subtle functional changes is limited, particularly in clinical trial settings where such changes are expected to be small. Alternative measures, such as timed performance tests and functional composites, have been proposed to address these limitations (16), but results have been mixed.

Over the last two decades, evidence has emerged supporting the role of the cerebellum in cognition and emotion, challenging the traditional view of the cerebellum as a structure solely dedicated to motor control (17, 18). One significant contribution to this shift in perspective was the identification of the Cerebellar Cognitive Affective Syndrome (CCAS) (19), a condition resulting from cerebellar damage and characterized by deficits in executive functions, spatial cognition, linguistic processing, and affect regulation (20). The identification of the CCAS likely contributed to increase interest in cognitive symptoms across cerebellar disorders, including FRDA. Indeed, in recent years, research has shown that, similarly to CCAS, FRDA patients also exhibit a range of cognitive impairments when tested for tools specifically designed to assess distinct cognitive functions, including information processing speed and executive function (4, 9, 21–24).

However, since cognitive impairments are not the primary concern for most FRDA patients, cognitive assessment is largely absent from clinical evaluations and rating scales (11). Most studies rely on traditional neuropsychological tools, such as the Montreal Cognitive Assessment (MOCA) and the Mini-mental State Examination (MMSE) to assess general cognitive status. In contrast, research identifying cognitive impairments in FRDA has employed more targeted tests, including the Stroop Test (25), the Simon Task (10), and the Wisconsin Card Sorting Test (26), among others (23). These tests commonly employ time-based quantitative measures, including reaction time (RT), movement time (MT), and other related metrics, which are particularly relevant for evaluating information processing speed and executive functions, where impairments are most consistently observed (22). The adoption of computerized tools in cognitive testing has enhanced the precision, objectivity, and replicability of these tests (27), facilitating the development of tailored tests targeting specific cognitive domains and patient populations.

To date, there is no cure for FRDA (28). Despite the growing number of clinical trials, the recently approved Skyclarys™ (Omaveloxolone) (29) is the only treatment shown to slow disease progression. Clinical trials primarily use clinical rating scales as endpoints, supplemented by timed performance measures and patient reported outcomes (PROs). Cognitive functioning is rarely evaluated, and speech impairments are also insufficiently addressed. Results from clinical trials have generally been disappointing. Possible explanations include short trial durations, small sample sizes, placebo effects, and the inherent limitations of clinical rating scales, particularly their lack of sensitivity to subtle functional changes (30).

This review examines the clinical trials conducted for FRDA, focusing on the instruments used to evaluate treatment outcomes, their limitations, and potential improvements. It briefly describes the epidemiology, pathology, and clinical features of FRDA. It explores the role of the cerebellum in motor control and cognition, highlighting dominant theoretical models. Tools used to assess FRDA patients are reviewed, revealing a predominant focus on ataxia symptoms while neglecting cognitive impairments. A dedicated section specifically reviews the research conducted on cognitive impairments in FRDA patients to date, with a focus on the type of tests used, particularly on those employing objective and precise time-based measures. It then evaluates completed and ongoing clinical trials, identifying trends and gaps in the assessment tools employed, which include almost exclusively three types of measures as endpoints to assess the clinical status of patients: clinical rating scales and, to a lesser extent, timed-performance and patient reported measures. Finally, it argues for the inclusion of well-established instruments, adapted to technological advancements, to obtain more sensitive and reliable evaluations of FRDA patients.

## 2 Friedreich ataxia

FRDA is the most common autosomal recessive form of ataxia. It has the highest prevalence in Europe, where it shows a negative gradient from west to east of the continent, with estimated values between 1:20000 and 1:750000, and carrier frequencies between 1:60 and 1:500 depending on the region (31, 32). Worldwide, it has an estimated prevalence of around 1:50000 among people with European ancestry. Data from other populations are thus far sparse, but FRDA seems to be restricted to individuals of European, Middle Eastern, North African, and Indian descent (33, 34). Male to female distribution is equal.

FRDA is caused by an expanded GAA trinucleotide repeat in the first intron of the Frataxin (FXN) gene. This gene encodes a protein called frataxin, required for the proper functioning of mitochondria. While the normal version of the gene contains 5–33 GAA repeats, in FRDA patients FXN alleles present between 66 and 1700, with most alleles containing between 600 and 1,200 repeats (33, 35, 36). In the vast majority of cases, FRDA is caused by homozygous GAA triplet expansions in the first intron of the FXN gene, but around 2–4% of the patients are compound heterozygotes for an abnormally expanded GAA repeat on one allele and another intragenic pathogenic variant, such as point mutation or a small deletion, on the other allele (35). The FXN mutation downregulates frataxin, which results in abnormal mitochondrial iron accumulation (36–38). This leads to abnormalities in a variety of structures, primarily the dorsal root ganglia and dorsal horns of the spinal cord, the dentate nuclei of the cerebellum, and the spinocerebellar and corticospinal tracts. While brainstem and spinal cord abnormalities seem to be linked to developmental deficits (39, 40), alterations in the cerebellum and cerebral cortex regions appear to follow a more progressive degenerative course (41–45). The length of the expanded repeats, and particularly the length of the shorter one, inversely correlates with age at disease onset, severity, rate of progression, disease duration, and age of death (36, 46–48).

FRDA is characterized by slowly progressive ataxia with a typical age of onset between 10 and 15 years of age (33). For most patients, symptoms start under the age of 25, but in a smaller number of cases it has a late (after the age of 25) or very late (after the age of 40) onset, the disease showing in these cases a slower progression (49). FRDA rate of progression is variable, and it has been found to be more rapid in individuals with earlier onset (50). The mean time until patients become confined to a wheelchair is around 10 years from symptom onset (36). Although it has increased over the last years, mean life expectancy is around 40 years, with cardiac failure as the most frequent cause of decease (1, 34, 48). FRDA neuropathology shows notable differences in the vulnerability of neuronal systems and in the timing when they become affected. Clinically, this translates into different timing and progression rate of cerebellar, proprioceptive, and pyramidal signs and symptoms, with patients showing the first symptoms when cerebellar signs appear and gradually developing a variety of pyramidal, sensory and cognitive symptoms as the disease progresses (4, 23).

FRDA manifests with a wide spectrum of ataxia and non-ataxia clinical features, including neurological and non-neurological signs and symptoms. The first overt symptoms typically include slowly progressive gait and limb ataxia, dysmetria, lower-limb areflexia, and dysarthria (51, 52). Other ataxia symptoms such as dysphagia, proprioceptive and superficial sensory loss, weakness and atrophy of the extremities (particularly the lower limbs), loss of muscle tone and spasticity gradually follow (42). The most frequent non-ataxia symptoms include abnormal eye movements, scoliosis, deformities of the feet, bladder dysfunction, cardiomyopathy, decreased visual acuity, hearing loss, depression and diabetes (47). While most early studies concluded that cognition was not affected in FRDA, more recent research have provided evidence of significant impairments in many cognitive domains, including attention, executive function, visuoconstructive and visuoperceptual abilities, verbal fluency, and social cognition tasks (23). These findings suggest that interruptions of the cerebro-cerebellar circuits may be functionally important in FRDA (52), in line with evidence accumulated over the last decades showing the involvement of the cerebellum in the organization of higher order functions beyond the motor domain (18, 53).

## 3 The role of the cerebellum in motor control and cognition

The pathology of FRDA is characterized by the degeneration of various structures traditionally associated with sensorimotor functions. This emphasis on motor symptoms initially led to an under appreciation of potential cognitive impairments in FRDA. However, in contrast with other structures predominantly associated with sensory and motor processing, the cerebellum is also critically involved in higher-order functions. Historically viewed as a structure primarily responsible for refining movement execution (54, 55), the cerebellum is now understood to play critical roles in cognitive, emotional, and autonomic regulation (53, 56–58). Its relative homogeneous cytoarchitecture and modular organization, coupled with cerebro-cerebellar connections mediated through the deep cerebellar nuclei in closed-loop systems, suggest that the cerebellum applies similar computational processes to distinct incoming signals. Functional differences across cerebellar regions largely arise from differences in connectivity, allowing the cerebellum to participate in motor, cognitive, affective, and vestibular functions (59–61). These computations are thought to involve the construction and refinement of internal models, which are continuously updated through error signals reflecting the mismatch between the intended and the actual outcomes (62). This optimization process enhances the speed, consistency, and appropriateness of responses (53) across domains. Consequently, damage to specific cerebellar regions leads to domain-specific motor, cognitive, affective, or vestibular deficits (63).

### 3.1 Cerebellum in motor control

The cerebellum supports motor control by maintaining internal models of motor dynamics, refined through experience and learning (60, 64, 65). These models include *forward models*, which predict movement outcomes, and *inverse models*, which generate the motor commands needed to achieve a desired state. Forward models use sensory feedback to predict system states and adjust motor commands when prediction errors (PEs) occur (62, 66, 67). As motor experience accumulates, these models are updated and refined through continuous error-based learning, which improves the precision of predictions over time (68). Inverse models, in contrast, generate the motor commands necessary to reach a target state, adapting through sensory feedback to maintain motor accuracy (60, 64). Both forward (69–71) and inverse models (72–74) are well-supported by evidence, though whether they operate independently or in parallel remains debated (64, 75, 76). Regardless, predictive error-based learning is widely recognized as fundamental to the coordination and precision of movement.

The role of the cerebellum in motor control is well illustrated by the cerebellar motor syndrome (CMS). The CMS manifests as ataxia, dysmetria, dysarthria, dysphagia, and tremor (20, 77), and results from cerebellar damage. The leading explanation for these deficits is the disruption of internal models, impairing the brain’s ability to generate and update accurate movement predictions (63). This leads to deficits in coordination, performance monitoring, and timing (78–81). Specifically, forward model disruptions cause inaccurate predictions, while inverse model disruptions lead to disorganized muscle activity, reducing motor accuracy (82, 83).

### 3.2 Cerebellum in cognition

Beyond motor control, the cerebellum plays a crucial role in cognition, language, emotion, and autonomic regulation (53, 56, 57, 84). Damage to the posterior lobe of the cerebellum has been associated, for example, to the cerebellar cognitive affective syndrome (CCAS), which, rather than encompassing motor symptoms, presents with deficits in linguistic processing, spatial cognition, affect regulation, and executive functions (19, 85). Just as CMS exemplifies cerebellar involvement in motor control, the identification of CCAS has provided key evidence for its contribution to cognition.

A dominant explanation for the cerebellum’s role in cognition relies on the concept of internal models and the associated notions of prediction and error-based learning, originally described for motor control (65, 71, 76, 86). According to this view, the cerebellum’s predictive functions have evolved to support higher-order cognition, including planning, attention, and working memory. Through its connections with frontal, prefrontal, and parietal cortices (60, 87), the cerebellum participates in predictive processes that optimize cognition in the same way it optimizes motor actions. This aligns with the central tenet of contemporary cognitive neuroscience, which suggests that the brain functions as a predictive machine, constantly making and adjusting predictions about future states of the environment to minimize PE (88, 89). Internal models underpin the cerebellum’s involvement in cognitive functions such as language processing (90–92), verbal working memory (93), social cognition and affective processing (62), pattern detection and sequencing (94), and executive functions such as planning and attention (60). These predictive mechanisms enable the cerebellum to contribute to performance monitoring and optimization across diverse tasks (83). Thus, the cerebellum plays a role not only in motor coordination but also in higher-order cognitive and emotional functions.

In FRDA, cerebellar pathology primarily affects the dentate nuclei, which serve as the primary output structures of the cerebellum (95). The dentate nuclei are highly organized, with distinct subdivisions receiving input from specific areas of the cerebellar cortex and projecting to defined regions of the cerebral cortex. This anatomical organization suggests that cerebellar dysfunction in FRDA arises from dentate damage disrupting precise cerebro-cerebellar connections. Since different subdivisions of the dentate nuclei relay information through functionally distinct cerebro-cerebellar loops, their degeneration can potentially affect multiple domains. Specifically, while the motor and premotor connections contribute to impairments in movement initiation, coordination, and fine motor control, the prefrontal and posterior parietal connections mediate higher-order cognitive functions, including executive function, attention, and working memory (84, 96–98). As a result, damage to the dentate nuclei in FRDA is likely to contribute to both motor and cognitive deficits, consistent with cerebellum’s known role in these domains.

It is important to note that while cerebellar involvement remains the most established hypothesis for the origin of cognitive deficits in FRDA, other mechanisms may also contribute. These include mitochondrial dysfunction related to frataxin deficiency, leading to impaired cellular energy metabolism and neural fatigue, and possible neurodevelopmental anomalies, given the early onset and slow progression of cerebellar and cortical changes in FRDA. Together, these mechanisms likely interact in a multifactorial manner to shape the cognitive profile observed in FRDA.

## 4 Assessment of ataxia symptoms in FRDA

FRDA diagnosis is established by genetic testing (33) following the observation of clinical, musculoskeletal, cardiac, perceptive and endocrinologic features, often supplemented by family history. After diagnosis, evaluations are recommended to determine the extent of the disease and the specific needs of the patient, and to assist with the management of the disease. A consensus document containing a series of clinical management guidelines^1^ was originally published in 2014 (99) and later updated in 2022 (100). The updated document expands on the original recommendations across multiple domains, including Neurology, Cardiology, Endocrinology and metabolism, Orthopedics and musculoskeletal, Genetics, Hearing, speech and swallowing, Vision, Sleep and fatigue, Pain and sensory symptoms Autonomic dysfunction, Psychosocial aspects and quality of life, Reproductive health and pregnancy, and Pharmacological and non-pharmacological interventions. The neurological components include ataxia, weakness, neuropathy, spasticity and muscle spasm, restless legs, mobility, dysarthria, dysphagia, vision, bladder function, bowel function, sexual function, audiological function, cognition, rehabilitation, pain, fatigue, and sleep (100). Each of these dimensions is evaluated using various instruments, detailed in the consensus document. This section focuses on the assessment of ataxia signs.

### 4.1 Clinical rating scales

Ataxia assessment is primarily based on clinical rating scales, which serve as the cornerstone for evaluating symptoms in both clinical practice and longitudinal studies. These scales are frequently used as primary and secondary endpoints in clinical trials, reflecting their importance in tracking disease progression and evaluating treatment efficacy. In addition to rating scales, assessments may also incorporate timed performance measures, functional composites, and PROs (101). The most commonly used scales are the International Cooperative Ataxia Rating Scale (ICARS), the Scale for the Assessment and Rating of Ataxia (SARA), and the Friedreich Ataxia Rating Scale (FARS) (102). While detailed descriptions and scoring information are available in comprehensive reviews (8, 11), this section briefly summarizes the structure and key features of these instruments to provide context for their application in FRDA research and care.

The ICARS, initially developed for cerebellar ataxia in clinical trials, was the first clinical rating scale accepted for FRDA assessment (103). It comprises 19 items across four clinical subscales: posture and gait disturbances, kinetic functions, speech disturbances, and oculomotor function. The scores of each scale can be summed to indicate the global severity of the syndrome, with completion taking 12–21 min.

The SARA was developed as a simpler alternative to ICARS. It was originally designed to measure cerebellar symptoms in spinocerebellar ataxia (14), and later validated for the assessment of other ataxias (15), including FRDA. It consists of eight items evaluating gait, stance, sitting, speech disturbance, finger chase, nose-finger test, fast alternating hand movements, and heel-shin slide. Scores are summed to indicate global severity, with completion by a trained health care professional taking <15 min. SARA focuses on cerebellar ataxia symptoms and excludes other neurological signs, which can be documented using the Inventory of Non-Ataxia Signs (INAS) (104, 105), including 16 non-ataxia items assessed semi-quantitatively (105, 106): areflexia, hyperreflexia, extensor plantar response, spasticity, paresis, amyotrophy, fasciculations, myoclonus, rigidity, chorea, dystonia, resting tremor, sensory symptoms, brainstem oculomotor signs, urinary dysfunction, and cognitive impairment.

Unlike the previous scales, the FARS was specifically designed and validated for FRDA assessment (107). Its original version includes a neurological examination (FARSn), a functional staging assessment evaluating overall mobility (functional disability staging, FDS); a patient-reported assessment of activities of daily living (ADL); and a series of timed measures of performance, including the PATA Rate Test (PRT) for speech, the 9-hole peg test (9HPT) for upper limb function, and a 25-foot timed walk (T25FW) to assess gait. FARSn assesses bulbar functions, upper limb coordination, lower limb coordination, peripheral nervous system, and upright stability. A modified version (mFARS) excludes non-ataxia items, focusing on bulbar functions, upper limb coordination, lower limb coordination, and upright stability. Completion takes 15–45 min depending on the version.

Over the years, SARA and FARS have largely replaced ICARS in FRDA assessment due to superior reliability and reduced ceiling or floor effects (12, 102, 108). Both scales correlate significantly and show comparable internal consistency and inter-rater and test–retest reliability (8, 11), and both continue to undergo refinements, including validation for video-based assessments (109), such as the SARA^home^ (110), for remote evaluations.

Despite their strengths, these scales have limitations. They are semiquantitative and inherently subjective, which limits their sensitivity and reproducibility (16). Additionally, they primarily focus on motor features of cerebellar dysfunction with limited attention to non-ataxia symptoms, and cognitive functions are notably underrepresented, reflecting their origins in an era with less understanding of the cerebellum’s role in cognition. Current knowledge has not been integrated into the clinical scales, and neuropsychological tools are used only in a limited number of studies. These limitations highlight the need for complementary tools to assess neurological impairments in FRDA.

### 4.2 Timed performance measures

Timed performance measures complement clinical rating scales, offering objective, quantifiable data. The FARS incorporates three such measures: the PRT, the 9HPT, and the T25FW. The PRT evaluates dysarthria and neuromuscular coordination by measuring how rapidly and clearly patients can repeat the syllables “PA-TA” within 10 s. It is repeated twice, with the total syllables counted providing a quantifiable measure of speech performance. It offers a simple, reproducible method to assess speech clarity and coordination (111). In the 9HPT, patients sit at a table with a container holding nine pegs and a block containing nine empty holes. They must place the pegs into the holes and remove them as quickly as possible, with two trials for each hand. The average completion time across trials is recorded, typically ranging from seconds to several minutes. Administered in under 10 min, the 9HPT assesses fine motor skills and has become a standard tool in FRDA research. In the T25FW, the patient is directed to one end of a marked 25-foot course and is instructed to walk to the other end as quickly as possible, but safely. Patients are allowed to use assistive devices. The time is measured from the initiation of the instruction to start and ends when the patient has reached the 25-foot mark. The task is immediately administered again by having the patient walk back the same distance. The score is the average of the two completed trials, which range from 4–5 s to several minutes. The task is administered in 1–5 min.

Timed performance measures offer high inter-rater reliability and less evaluator bias than clinical rating scales, making them valuable for detecting subtle disease progression (11, 107). Some findings suggest that, when performance in the 9HPT, the T25FW, and the Sloan Low Contrast Letter Chart (SLCLC), a measure of visual acuity not included in the FARS are combined to create composite scores, these may more accurately reflect disease progression than single measures or FARSn (16). Compared to clinical rating scales such as FARSn, composite scores are more objective, provide higher inter-rater and test–retest reliability, are quicker to administer, require minimal training, and allow for new tests to be added easily (101). As a downside, these tests also present some specific constraints, particularly a prominent floor effect in patients suffering from severe FRDA. This becomes particularly obvious in the case of the T25FW, which is only useful in patients that are not confined to a wheelchair yet which, according to the literature, happens to most patients after 10 years from disease onset, on average. Other limitations, such as variations in the command for walking, which may affect the reliability of the measurements, or the difficulty to observe gait features affecting performance (gait deviations, ability to adjust gait, impact of endurance on gait) have also been pointed out (112).

Clinical rating scales and timed performance measures remain pivotal for FRDA assessment, providing robust tools to evaluate neurological symptoms, track progression, and assess therapeutic efficacy. However, these tools focus predominantly on motor and cerebellar dysfunction, leaving cognitive impairment either unaddressed or assessed in a coarse and limited manner. While the SARA-associated INAS offers a basic four-point scale for cognitive assessment, it lacks the granularity needed to fully capture the cognitive challenges that patients may face. Furthermore, the full version of the FARS does not include specific measures for cognitive function. In the next section, we will explore the current understanding of cognitive impairment in FRDA and the tools used for its assessment.

## 5 Cognitive impairment in FRDA

Cognition in FRDA has received less attention than gait, movement and sensory symptoms, despite well-documented evidence of reduced information processing speed (IPS) that cannot be explained by motor difficulties or ataxia alone (24, 113–116). This oversight stems from the traditional focus of behavioral neurology on task-specific domains (117), where the cerebellum was long considered to function exclusively in motor control (103, 118). Consequently, neurological evaluations in FRDA have prioritized motor impairments, overlooking cognitive deficits. Furthermore, motor, speech, and sensory impairments in FRDA can confound cognitive assessment by affecting reaction times, speech fluency and comprehension, leading to an underestimation of subtle impairments when using general screening tools (4).

Growing recognition of the cerebellum’s integral role in cognitive and affective functions (18, 77, 119–122) has spurred interest in the cognitive aspects of FRDA. To explore the cognitive assessment tools used in research (Anytime to July 2024), we used the Pubmed database^2^ to conduct a search (keywords: Friedreich ataxia + attention, cognition, executive, language, memory, neuropsychology, processing speed, reaction time). Thirty-four studies, summarized chronologically in Supplementary Table 1, were identified, with 26 published since 2010. These studies employ a range of tools, including general cognitive screening instruments, intelligence tests, neuropsychological tests, and ad-hoc designed domain-specific tools, reflecting increasing interest in understanding the cognitive profile of FRDA.

**Figure 1:**
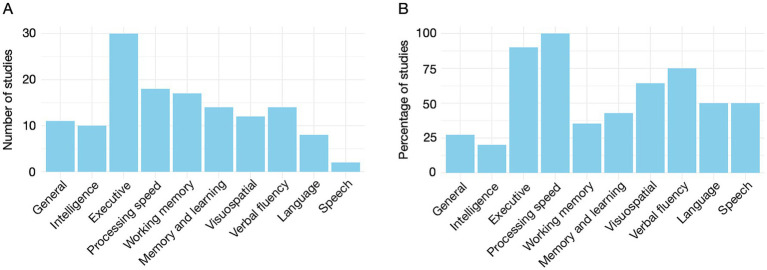
**(A)** Number of studies employing instruments dedicated to the assessment of the general cognitive state, intelligence, and a series of cognitive dimensions. **(B)** Percentage of studies reporting significant results among those evaluating each particular dimension.

**Figure 2:**
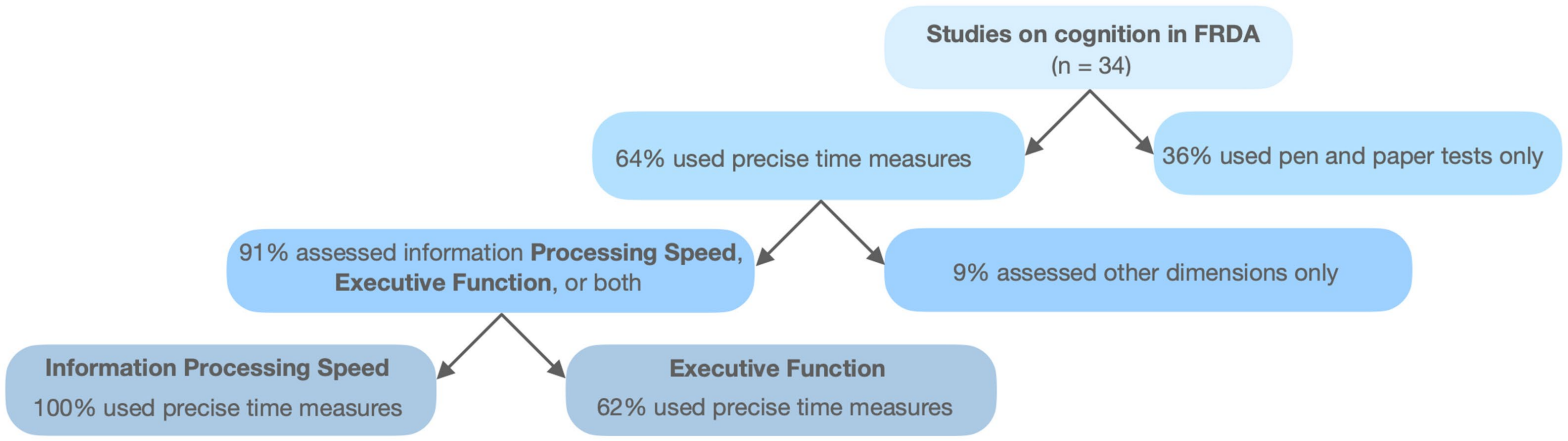
Flowchart representing the use of precise time measures and computerized methods in studies on cognition in FRDA patients.

Figure 1 synthesizes data from Supplementary Table 1. General cognitive assessments show limited sensitivity in detecting FRDA-related impairments, while domain-specific tools consistently reveal significant impairments. While a detailed review of the cognitive profile in FRDA is beyond the scope of this article, a systematic review and meta-analysis by Naeije et al. (23) provides a comprehensive summary, confirming deficits in language, attention, executive function, memory, and visuospatial perception. Unlike that review, we consider basic information processing speed (IPS), understood as a measure of speed and efficiency of fundamental processing stages, as a specific dimension in the present work. As Figure 1 shows, executive function is the most frequently assessed domain, with impairments reported in 90% of studies. IPS, however, stands out, as all studies assessing it report significant deficits. Importantly, IPS is assessed using precise (millisecond-level) time measurements obtained via electronic or computerized tools (Figure 2). These methods are also commonly used for executive function and have demonstrated high sensitivity in identifying deficits. In the following sections, we focus on (a) the use of precise timing and (b) computerized methods for cognitive assessment.

### 5.1 Use of quantitative time measures for cognitive assessment

Initial indications of cognitive impairment in FRDA patients arose from observations of a general IPS slowdown beyond what motor deterioration alone can explain (113). IPS is typically measured using mental chronometry, a technique formally introduced in the late 19th century and still central in neuroscience today (123). By examining the timing of mental operations, this approach connects task performance to neural efficiency and cognitive integrity, providing detailed insights into sequential processing stages. Furthermore, the objectivity and reproducibility of RT-based test make them particularly suited in detecting and monitoring cognitive deficits in neurological conditions such as FRDA, where cognitive impairments are often reflected in slower or variable RTs. As Figure 2 shows, most of the studies included in Supplementary Table 1 employ some type of precise time measurement to assess some aspect of cognitive functioning, typically basic IPS or executive function.

#### 5.1.1 Basic IPS

Basic IPS is typically evaluated with tools such as Simple Reaction Time (SRT) and Choice Reaction Time (CRT) tasks. Two measures are usually considered in these experiments: RT, i.e., the time taken to react to a stimulus and initiate a response; and Movement Time (MT), the time taken to complete the response. It is assumed that RT involves aspects of cognition and assess the speed and efficiency of information processing, while MT reflects motor and coordination components (124, 125). Two studies (113, 115) using precise electronic timers reported longer RT and MT in FRDA patients compared to controls, indicating a general processing slowdown. Six additional studies employed the Reaction Unit of the PC-Vienna System (126), a computerized implementation of SRT and CRT tasks. Two of these (116, 127) reported significant deficits in RT and MT in SRT and CRT in FRDA patients. Another study identified impairments in RT and MT in both tasks (26), while in late-onset FRDA, deficits appeared primarily in MT for CRT tasks while in late-onset FRDA, deficits appeared primarily in MT for CRT tasks (128). An additional study linked depressive symptoms, assessed via the Beck Depression Inventory, to RT performance in CRT tasks (129). Finally, a longitudinal study found progressive deterioration in RT and MT over time in SRT and CRT tasks (22). In sum, precise electronic timing consistently demonstrates sensitivity in detecting IPS deficits in FRDA patients.

#### 5.1.2 Executive function

Executive function is the most commonly cognitive domain in FRDA studies (Figure 1). Classical tests, such as the Go/No-go, Simon, and Stroop tasks, where time-based measures are central, have consistently revealed impairments of executive function in FRDA (10, 22, 24, 25, 115, 130–135). Computerized versions of these tasks have been particularly effective.

Besides studies addressing executive functions in FRDA patients with classical neuropsychological tests, a number of studies have utilized RT-based measures to explore motor planning, control, and interference in FRDA. Corben et al. (118) examined movement reprogramming by requiring participants to adjust reciprocating movements on a tapping board in response to an oddball stimulus. RT and MT were measured, revealing impairments in movement preparation and execution in FRDA patients, with a negative correlation between age of onset and reprogramming conditions, suggesting effects on motor planning development. Using a similar device, the same group found that FRDA patients did not benefit from high visual cue levels during sequential movement planning, unlike controls, further implicating motor cognition development. A subsequent study using a computerized version of the Fitts’ task identified disproportionately prolonged preplanning and terminal accuracy phases, reflecting impaired access to prefrontal regions critical for movement preplanning and online error connection (136).

In parallel, precise timing measures in three studies used eye movement markers to explore the cerebellum’s role in cognitive control. One study (137) found significant differences in antisaccade and memory-guided saccade latencies, supporting eye movement markers as reliable biomarkers in FRDA. Another (138) examined attentional orienting using the gap overlap paradigm, finding FRDA-related deficits in disengaging attention due to cerebellar involvement. A final study (139) used a saccade reprogramming task, showing FRDA patients had greater latency increases and lower accuracy when responding to unexpected changes, interpreted as disruptions in cerebellar-cortical connectivity.

### 5.2 Use of computerized tests for cognitive assessment

RT measures provided some of the earliest empirical evidence for the temporal structure of mental operations, and their utility has only grown with advances in computerized testing. Today, precise time measurements, often recorded at the millisecond level, enable researchers to dissect the subtleties of cognitive impairments, including those observed in FRDA.

Most FRDA studies in Supplementary Table 1 have used traditional, printed versions of common neuropsychological tests, but 10 studies employed computerized versions. These include tools assessing visuospatial and visuoconstructive functions [Judgement of Line Orientation Test (JLO), and the Facial Recognition test (FRT)] (116), attention [Test of Attention Performance, Continuous Performance Test (CPT)] (22, 24, 26, 128); and executive function (Tower of London, N-back, Stroop, Go/NoGo, and Simon task) (10, 24, 130–133, 140). In addition to those studies, six works (22, 26, 116, 127–129) used SRT and CRT, implemented in the Reaction Unit of the PC-Vienna System, to assess IPS. IPS was also evaluated through computerized methods in three other studies (113, 115, 132). In one study a computerized finger tapping task was also used (130). Finally, all the instruments designed *ad hoc* to address specific purposes were computerized tools (26, 114, 118, 128, 130, 136–139, 141, 142). With the exception of the JLO, the CPT, the Tower of London, and the N-back task, the computerized tools used for the cognitive evaluation of FRDA patients were successful at detecting significant performance differences between patients and controls or showed sensitivity to detect changes over time in patients’ performance (22).

The advances in digital technologies have led to computerized versions of many tests commonly used in neuropsychological evaluation, Cognitive Psychology, and Cognitive Neuroscience. These tests can be tailored for specific research purposes and are often integrated in commercial platforms such as the Penn CNB, the Cogstate Cognitive assessment System, the Pearson’s Q-interactive platform, or the Cambridge Neuropsychological Test Automated Battery (CANTAB). However, most studies evaluating cognition in FRDA patients have relied on traditional tests, where scores are typically based on response correctness or accuracy and on the quality of the responses, using variables such as the number of correct answers and errors to a series of items (e.g., JLO), the length of the longest correctly repeated sequency (e.g., Digit Span), the number of items correctly recalled (e.g., RAVLT), or the number of words correctly produced (e.g., FAS test), for example. In a smaller number of tests [e.g., Trail Making Test (TMT), Tower of London, Tower of Hanoi, Attention Matrices, Stroop], the time taken to complete a given task, usually in the range of seconds or minutes, is also considered as a variable. Digital tests have the advantage of incorporating precise time measures, complementing the information provided by their traditional versions. Indeed, all the digital versions of the tests mentioned above, with the exception of the JLO, the FRT, and the Tower of London, use precise time measures as an outcome.

To summarize, research on cognitive function in FRDA has steadily advanced over the last 15 years. While many studies still use traditional neuropsychological tests, an increasing number employ computerized tests and precise time measures. This trend reflects the need to evaluate deficits in IPS and executive functions, consistently observed in FRDA patients, and to align with theoretical and technological advancements in neuropsychological evaluation. Unfortunately, contrary to motor symptoms, the evaluation of cognition in FRDA is to date usually not implemented in general clinical practice nor in clinical trials.

## 6 Outcome measures in clinical trials on FRDA

Supplementary Table 2 summarizes the interventional clinical trials on FRDA, registered on www.clinicaltrials.gov, a database maintained by the National Library of Medicine of the US, and on www.clinicaltrialsregister.eu, the EU Clinical Trials Register. These databases collectively list 90 entries, including various phases of the same studies conducted over the past two decades. Many trials are completed, others are still ongoing, while others have yet to start.

A total of 72 interventional trials are reported in Supplementary Table 2, or 54 when grouping together corresponding to different phases or extensions of the same study. Of these, 35 (#1–5, 7–21, 23, 24, 26–29, 31, 33–35, 38, 41, 45, 49, 51) include some form of neurological assessment as primary or secondary outcome. Trials without neurological assessments are either trials target non-neurological symptoms (most often cardiac) or early phase trials just aiming at determining the safety and tolerability of the medicaments. Note however that the use of neurological indices in early phases of many studies is not uncommon.

**Figure 3:**
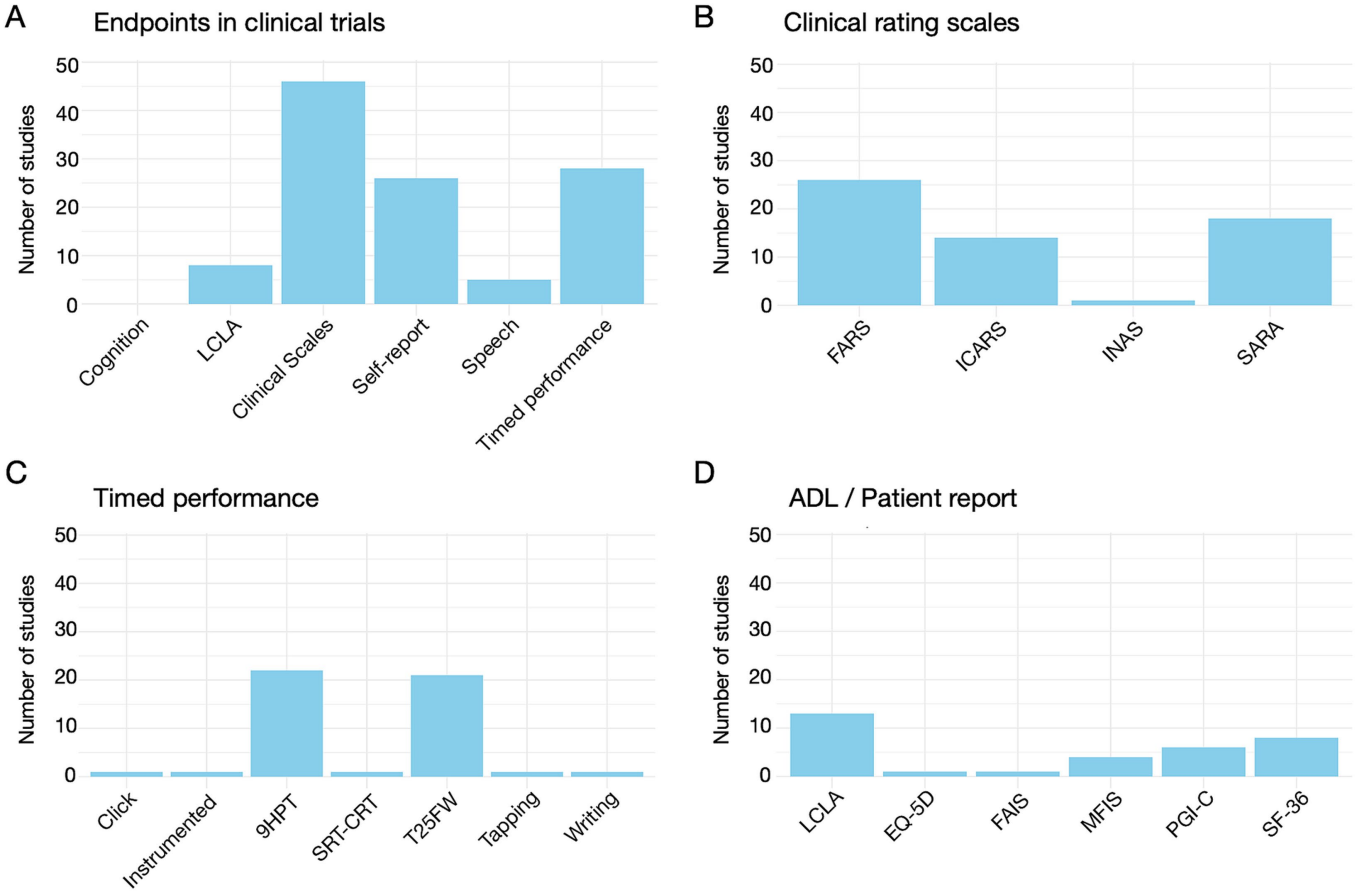
**(A)** Number of studies using each type of endpoint. **(B)** Number of studies using each of the different clinical rating scales. **(C)** Number of studies using each of the timed performance measures. **(D)** Number of studies using each of the different ADL/Patient report scales (LCLA, Low Contrast Letter Accuity; EQ-5D, EuroQol 5-Dimension; FAIS, Friedreich Ataxia Impact Scale; MFIS, Modified Fatigue Impact Scale; PGI-C, Patient Global Impression of Change; SF-36, Short Form-36 Health Survey).

For the goals of this review, three main conclusions can be drawn from Supplementary Table 2. Figure 3 summarizes de information contained in that table. Three main conclusions can be extracted from that figure. First, almost all trials using neurological assessments (all but one) rely on clinical rating scales, either as primary or secondary outcomes. Timed performance measures, most commonly the 9HPT and the T25FW or related tests, and PROs and/or ADL are also common. Second, specific speech assessments are rarely included as outcomes. Third, cognitive evaluations are absent from all trials.

While some of the clinical trials listed in Supplementary Table 2 lack posted results in the databases, many are available in peer reviewed publications. However, not all results align with initial trial proposals, as some studies report unlisted outcomes or exclude specified ones. Additionally, several trials are absent from the clinical trial databases. To address this, we extended our search to PubMed (see text footnote 2) with the keywords “Friedreich Ataxia,” identifying 100 relevant publications, many referring to trials already included in Supplementary Table 2. We summarized in Supplementary Table 3 27 additional articles on clinical trials which (1) use any kind of neurological evaluation (2) include exclusively or mainly FRDA patients, and (3), are not included in Supplementary Table 3 (although in some cases are related to the clinical trials included in Supplementary Table 3). A review of Supplementary Table 3 yields conclusions similar to Supplementary Table 2. Nearly all studies, except for two older ones use clinical rating scales as outcome measures. These exceptions focus on RT tasks (143, 144). Five studies include timed performance measures like the 6MWT, the 9HPT and related tools. Three clinical trials incorporate ADL and/or PROs. None evaluate speech or cognitive functioning as clinical outcomes.

Clinical interventions are grounded on the rationale that a given treatment, aimed at modifying specific biological or biochemical parameters (e.g., frataxin levels, oxidative stress indices), would induce measurable neurological effects. The neurological effects are measured in almost all cases by setting one or several clinical rating scales as primary or secondary outcomes, often in combination with timed performance measures, functional composites, and other instruments such as ADL and PROs. As can be seen in Supplementary Tables 2, 3, a number of clinical trials have reported improvements in clinical rating scales scores, including dose-dependent improvements (145–147), while others found no significant impact (148–165). Importantly, when biomarkers and clinical rating scales scores are both included as endpoints, an examination of the correlation between the effects of the intervention on each of these variables would be expected. However, such analyses were quite rarely found, in some cases due to the small samples and the short duration of the treatments (166), and in others because the results failed to show significant effects of the interventions on the biomarkers, on the clinical scales scores, or on both. Still, even when effects on both variables are observed, the statistical correlation between those effects is not always investigated or reported (167). In sum, only nine of the clinical trials included in Supplementary Tables 2, 3 found significant effects on both variables (145, 166–173), and only six of those trials performed such analyses, with three finding significant correlations (169, 170, 174), and three failing to statistically relate the effects on the biomarkers to those on the scales’ scores (168, 171, 172).

A broader review of FRDA trials highlights the generally poor results achieved so far. Several factors are pointed out in the literature as possible explanations (175, 176), from the simple lack of therapeutic activity of the treatment (30, 148), the insufficient understanding of the mechanism of action of the drugs tested (177), or the wearing off of the effects of the therapeutic agents (30), to generic problems such as potential practice or learning effects affecting the changing rate of the scores obtained during the assessment (145, 160, 176). Among the factors potentially affecting the lack of results, the most commonly cited are probably the use of small samples and the short duration of the clinical trials. Clinical trials in FRDA convey some difficulties related to its condition as a rare disease. Small patient populations, along with heterogeneity in age at onset, GAA repeat length, biomarker levels, symptoms, and progression rates, challenge recruitment and analysis and, consequently, the results and the conclusions that can be extracted (178). Short trial durations exacerbate these issues, often yielding “improvement trends,” or “improvements that did not reach statistical significance,” which may be a reflection of these limitations, but also an indication of the lack of sensitivity of the measures used. Other issues frequently reported as problematic in this regard are the open-label nature of many studies (i.e., patients know the treatment they are receiving), which makes it hard to discard placebo effects (179), and issues associated to the use of cross-sectional data from cohorts of patients in natural history studies instead of placebo groups, since it has been shown that these groups behave differently in clinical trials (147). An especially critical limitation lies in the type of measures used to evaluate treatment efficacy. These can be grouped into three primary issues: reliance on subjective, semiquantitative clinical rating scales and PROs, the use of low sensitivity timed-performance measures; and the absence of cognitive assessments, particularly for speech and executive function. The following sections will explore these limitations in detail.

### 6.1 Clinical rating scales

Clinical rating scales remain central to FRDA clinical trials for assessing neurological symptoms. FARS and SARA have largely replaced earlier instruments such like ICARS, with the modified FARS (mFARS) being selected as a primary endpoint in the MOXIe trial that led to the approval of Skyclarys™ (Omaveloxolone) (29). While mFARS has gained regulatory acceptance and is supported by extensive natural history data, it has not yet undergone formal psychometric validation, highlighting the ongoing need to rigorously evaluate the measurement properties of widely used scales.

Importantly, SARA, FARS and mFARS have been used to track neurological progression in two large natural history studies: the European Friedreich Ataxia Consortium for Translational Studies (EFACTS) (50), and the Friedreich’s Ataxia Clinical Outcome Measures Study (FACOMS) (180). These studies confirmed the scales’ suitability as clinician-reported outcomes in clinical trials (181). Moreover, EFACTS and FACOMS deepened understanding of the stage-depending progression of FRDA, identified differential severity and progression profiles based on age at onset and the length of the shorter GAA triplet expansion, and provided robust data for calculating the sample size needed for these scales to effectively detect changes in patients’ state over time (180, 182, 183). While the rating scales themselves have not changed substantially, the design of clinical trials around them has matured significantly. The availability of natural history data has helped clarify the limited success of earlier clinical trials and has laid the groundwork for more sensitive, targeted, and statistically powered studies moving forward.

Despite their strengths, clinical rating scales still present limitations (16, 146, 153, 184), particularly regarding their sensitivity at early and advanced disease stages (152, 159, 181, 185), and their focus on motor symptoms limits assessment of non-motor domains affected by FRDA, potentially overlooking relevant therapeutic effects (153). These challenges underscore the need for complementary, objective, and domain-specific outcome measures to improve the evaluation of treatment efficacy.

### 6.2 Timed performance measures

Timed performance tests like the T25FW and 9HPT widely used in FRDA clinical trials to address some limitations of clinical rating scales. These tests offer greater objectivity, precision, and resistance to evaluator bias, with strong inter-rater reliability, and are arguably more precise and sensitive to small changes in the functional state of the patients (11, 101).

While a valuable complement to clinical rating scales, timed performance measures also have notable shortcomings. These limitations arise from the complexity of the, the scoring time scale, and the small number of data points collected during each administration. First, the complexity of tasks, which involve multiple subprocesses, can obscure changes affecting only specific components of performance. Second, the long duration of trials, ranging from seconds in T25FW to minutes in 9HPT, exposes scores to uncontrolled variables, complicating detection of small changes. Finally, the limited data points collected per administration (two for T25FW and two per hand for 9HPT) reduce sensitivity and precision, making it harder to detect subtle changes in disability. Consequently, significant performance shifts may be required to detect therapeutic effects. The combination of these limitations affects the sensitivity, precision, and reliability of the measurements obtained. As a consequence, relatively big performance shifts may be required for these tools to detect any therapeutic effects.

In clinical trials, these tools have yielded poor results, with most studies reporting no significant effects on 9HPT or TF25FW (29, 147, 158, 172, 184, 185). Even in rare cases where effects were observed, these findings were not supported by other measures such as biomarkers or clinical scales (158, 161). Consequently, while timed performance measures offer greater objectivity than rating scales, their limited sensitivity and precision reduce their utility in FRDA trials.

### 6.3 Patient reported outcomes

PROs serve as secondary endpoints in many clinical trials, aiming to capture the patients’ perspective on the effects of a treatment. These measures reflect aspects not adequately assessed by clinical scales, such as fatigue, emotional wellbeing, social participation, and communication difficulties. Such symptoms can significantly impact patients’ ability to work, study, and maintain relationships, making them central to overall health and wellbeing.

PROs help to broaden the scope of assessment by highlighting symptom domains that may otherwise be overlooked. However, they are not without limitations. Generic (non-FRDA specific) PROs are often affected by significant ceiling and floor effects and may show poor responsiveness to change over time, reducing their sensitivity to detect meaningful treatment effects (186, 187). As a result, they may fail to fully capture the burden of disease or improvements relevant to patients with FRDA (188).

To address these challenges, disease-specific PROs have been developed. The Friedreich Ataxia Impact Scale (FAIS) was the first disease-specific PRO for FRDA and remained the only such measure for nearly two decades. While it has helped shape the understanding of patient experience in FRDA, concerns have been raised about its limited responsiveness to change, which may restrict its utility in interventional clinical trials (188). More recently, the Friedreich’s Ataxia-Health Index was introduced as a comprehensive, FRDA-specific measure of patient-perceived health status (189). While initial reports indicate high internal consistency and test–retest reliability, further evaluation is needed to support these findings and determine its sensitivity to therapeutic interventions and change over time.

In summary, while PROs may have certain methodological limitations, they remain a valuable component of the outcome framework in FRDA clinical trials. Importantly, regulatory authorities such as the US Food and Drug Administration emphasize the importance of incorporating meaningful endpoints, reflecting real-world impacts of disease and treatment on everyday functioning (190). This patient-centred approach aligns with broader shifts in clinical research toward outcomes that matter most to patients. In this context, PROs serve as a critical complement to clinical and biomarker-based assessments. As such, they enrich trial design by offering a more holistic view of therapeutic benefit, aligning more closely with patients’ lived experiences and priorities.

### 6.4 Assessment of speech and cognition

The lack of attention to speech and cognitive functioning in FRDA clinical trials is notable, given the cerebellum’s known role in cognition and growing evidence of cognitive impairments in FRDA patients. Dysarthria, a common symptom of FRDA, is rarely evaluated comprehensively, and cognitive assessments are virtually absent. While some clinical studies report treatment effects on speech capabilities, these findings are often underexplored due to the inadequacy of current clinical scales to address these domains, leading the authors to emphasize the need for future trials to incorporate comprehensive speech assessments (146, 176, 184). This omission is particularly significant, as speech and cognitive impairments have profound effects on intellectual and social development, autonomy, and overall quality of life (4). Addressing these dimensions is essential for understanding the broader impacts of treatments on FRDA patients.

In summary, while clinical rating scales, timed performance measures, and PROs each have methodological limitations, they remain essential components of outcome assessment in FRDA clinical trials. Although the scales themselves have not evolved substantially, significant progress has been made in understanding the strengths and limitations of the clinical scales, leading to improved use in trial design, particularly through insights gained from large natural history studies. Timed performance measures continue to provide objective and functionally relevant data, even if their sensitivity can be limited. PROs, despite challenges in responsiveness, offer valuable insights into the patients’ lived experience and their importance is increasingly recognized by regulatory authorities as meaningful endpoints. Future approaches should aim to enhance these tools with complementary, sensitive, and domain-specific measures, especially in underexplored areas such as speech and cognition. Current technological developments can complement traditional tools by providing a nuanced evaluation of motor and cognitive changes. Such advancements are crucial for minimizing the risk of false negatives and ensuring that therapeutic benefits are accurately captured (11, 191).

## 7 Alternative measures and current developments

The limited success of existing assessment tools in FRDA clinical trials underscores the pressing need for more accurate, objective, and sensitive measures. Research demonstrates that cognitive impairments in FRDA patients can be detected using both classical and modern cognitive tests. However, cognition remains largely unaddressed in clinical trials, often relying on superficial evaluations with general screening tools or self-reports. Integrating robust cognitive assessments into clinical trials, alongside advances in technology and experimental research, could address many current limitations.

### 7.1 Time-based measures

Tools from Cognitive Psychology and Neuroscience, such as RT-based measures, offer a valuable complement to traditional FRDA assessments. RT-based measures have been historically underutilized in clinical settings in favor of more complex tools believed to correlate better with everyday functioning, despite lacking alignment with current knowledge and theories of cognitive neuroscience (192, 193). RTs, however, are reliable indicators of central nervous system integrity (194). They enable the evaluation of specific cognitive and motor subcomponents by measuring the speed and efficiency of information processing through tailored task manipulations (22, 113, 132).

RT-based measures offer several distinct advantages. These tests are standardized, ensuring accuracy, reproducibility, and comparability across trials. Their objective assessments of symptom severity and treatment response bypass the limitations of subjective ratings and self-reports. The millisecond-level precision of RT measurements, combined with trial-by-trial accuracy tracking, allows for high sensitivity in detecting subtle changes. Existing research underscores their ability to detect small differences in healthy individuals and to monitor changes resulting from experimental manipulations. From a practical perspective, RT-based measures are cost-effective, requiring minimal resources, training, and expertise. They are quick to administer, enabling frequent testing and larger sample sizes at a reduced cost. Importantly, these measures can be adapted for use in specific patient groups, such as those in advanced disease stages, who may struggle to complete more demanding tests like the 9HPT and the T25FW (147). This adaptability, combined with their sensitivity and practicality, highlights the potential of RT-based measures for use in clinical trials.

In addition to RT tests, precise time-based tools are central to instruments such as the finger- tapping tasks, which offer similar advantages and are well-suited for assessing motor and cognitive functioning in patients with FRDA. These tasks, available in various formats, evaluate motor and cognitive functions, including psychomotor speed, hand dexterity, and timing control (195, 196). Depending on the version employed, performance metrics such as tapping rate, variability, amplitude, inter-tapping intervals, and accuracy can be assessed. Deficits in finger-tapping tasks are consistently observed in patients with cerebellar lesions (197), showing the cerebellum’s critical role in timing operations, such as movement timing (117), temporal prediction (198), sensorimotor synchronization (199), and coordinated eye and hand tracking (200). Precise timing control is essential for the coordination and execution of skilled movements, and deficits in this area may significantly undermine motor performance in FRDA patients. Finger tapping tests have been widely used in neuroimaging research to study brain activation patterns in FRDA patients compared to controls (3, 130, 201–203), consistently revealing significant impairments in patients. Recently, these tasks have been proposed as potential outcome measures for clinical trials (202), highlighting their utility in capturing critical aspects of motor dysfunction and their responsiveness to intervention effects.

A major limitation of clinical rating scales is their reliance on complex behaviors, where poor performance may stem partly from cognitive deficits. These scales produce semiquantitative scores that are subjective, imprecise, and unable to separate motor and cognitive subcomponents, which may be differently affected by FRDA. Timed performance tests face similar challenges in isolating specific impairments. In contrast, RT-based tests commonly used in cognitive neuroscience offer a more targeted approach. These tests employ simple, well-defined tasks grounded in theoretical and experimental research, manipulating one or a few variables in a controlled manner. This allows researchers to analyse distinct constructs individually or in combination and to test specific hypotheses. Clinical trials, where treatment effects are typically small and hard to detect in complex behaviors, could benefit from this approach by adapting existing tests or developing new tools based on similar principles.

### 7.2 Assessment of speech

Speech assessment in clinical trials is often overlooked, despite dysarthria being a hallmark symptom of FRDA. This oversight appears to mirror standard clinical practice, where therapeutic interventions for language impairments are largely overlooked (204). Addressing this gap is essential, as speech difficulties significantly impact communication, social interaction, and overall quality of life. Subtle changes in speech might appear even before disease onset, as it has been described in other types of ataxia (205). Dysarthria symptoms progress over the course of the disease, which makes speech a potential source of sensitive parameters for tracking clinical changes over time, both due to the natural progression of the disease and to the hypothetical effects of therapeutical interventions (206). Some clinical trials have actually reported positive effects of the treatment under study on speech abilities (146, 176, 184).

Although speech-related items are included in clinical rating scales, these tools have limited sensitivity to detect small performance changes. A few trials have used standalone speech assessments, but these too often rely on subjective evaluator judgement (152, 184), limiting both their sensitivity and objectivity. In contrast, acoustic speech analysis offers an increasingly viable alternative. By extracting quantitative features from recorded speech, these methods enable objective, comprehensive, high-resolution tracking of subtle changes over time. Over the past two decades, acoustic analysis has been successfully applied in other neurological conditions, including Parkinson’s disease, multiple sclerosis, and amyotrophic lateral sclerosis (207–212), and has shown similar promise in ataxias (213–216). A significant step forward has been the recent publication of recommendations by the Ataxia Global Initiative Working Group on Digital-Motor Markers, which outline best practices for quantitative speech assessment in clinical trials (217). These guidelines include considerations for hardware and software selection, task design, relevant speech features, and data analysis strategies. They represent a critical milestone toward standardizing and validating digital speech biomarkers in ataxia, with direct implications for their integration into FRDA trials.

Acoustic analysis measures have been successfully applied to speech evaluation in FRDA patients, identifying features that track disease progression and dysarthria severity (206, 218–223). The objectivity and sensitivity of these measures have led to their proposal as clinical endpoints in FRDA (206). However, despite this promise only a single trial to date has formally adopted acoustic speech analysis as an outcome measure (167). This highlights a significant gap between current research capabilities and their application in therapeutic development.

Given the growing evidence base and the availability of structured expert recommendations, integrating acoustic speech measures into future clinical trials represents a promising path forward. These tools offer greater accuracy and objectivity than traditional assessments and are better suited to capturing treatment effects over time.

#### 7.2.1 Digital implementation of assessment tools

The great versatility of digital tools enables researchers to implement virtually any existing cognitive assessment test into a digital form. Digital versions offer additional advantages, ensuring standardized, consistent testing conditions and thereby improving the precision and objectivity of performance measures. Automated delivery of task instructions and real-time feedback minimizes evaluator influence, enhancing reliability. Furthermore, automation of recording, scoring, and data storage streamlines processes, saving time and resources while improving cost-efficiency (192). These tools also allow trial-by-trial data collection, incorporating modalities like voice and video recording, and can be tailored to meet specific evaluation needs. Critically, digital tools enable testing beyond traditional research or clinical settings, including in patients’ homes, facilitating broader accessibility and flexibility. These characteristics make digital tests well-suited for tracking subtle changes in cognitive functioning over time, an aspect particularly interesting for their potential use as endpoints in clinical trials.

Efforts to develop remote evaluation tools for FRDA have gained momentum, particularly in response to the COVID-19 pandemic. For instance, studies have shown that assessments using mFARS and SARA scales can be reliably conducted via video conferencing platforms (109). Similarly, the SARA^home^ protocol demonstrated the feasibility of home-based ataxia assessment using tablet cameras, which offline performance ratings by evaluators (110). Beyond pandemic-driven needs, there is a growing demand for tools enabling remote, unsupervised patient assessments for clinical practice and for their potential use as endpoints in clinical trials. Such tools could provide continuous data on daily or even intra-day symptom fluctuations, improving cost-efficiency, enhancing clinical decision making, and supporting individualized care (224, 225). Systematic validation of these digital devices, variables, and algorithms should be prioritized in future research (226).

The development of such home assessment measures for use as endpoints in clinical trials has been pointed out by the US Food and Drug Administration as a goal to be achieved within the Clinical Trials Transformation Initiative (CCTI).^3^ Advances in web-based testing platforms present a promising avenue, offering secure data collection, encryption, and storage. These platforms expand scalability and enable the creation of large, detailed databases, potentially driving new insights across research domains. In Cognitive Psychology and Cognitive Neuroscience, web-based testing is rapidly gaining traction, with growing validation of classical tests and evidence supporting the precision of digital measures (227–230). Data accumulation is further accelerated by wearable and portable devices (192, 225), along with instrumented measurements (231). In the specific case of FRDA, the Ataxia Instrumented Measure-Spoon (AIM-S) exemplifies the potential of such technologies. This tool measures upper limb ataxia during the pre-oral phase of eating using wireless motion capture and advanced signal analysis algorithms. A recent study has demonstrated that the AIM-S provides more accurate and consistent measures of upper limb function than existing measures like the 9HPT (232). It is currently being used in a large observational clinical trial (233). Similarly, another ongoing observational clinical plans to conduct multiple home assessments of FRDA patients, with a particular focus on speech, via a mobile-health app (234). Looking ahead, advancements in machine learning and artificial intelligence are expected to further enhance data analysis, uncovering new insights to refine clinical evaluations and develop more precise trial endpoints (225, 235, 236).

Despite the ubiquity of digital technologies and their potential benefits, their integration into clinical trials remains limited. This is particularly evident in trials for FRDA, where endpoints for evaluating disease symptoms often rely on subjective, imprecise instruments with limited sensitivity to changes. In contrast, biological markers of treatment effects are measured using sophisticated and precise laboratory and neuroimaging techniques. Bridging this disparity by incorporating precise, objective digital measures into clinical assessments offers significant opportunities for developing novel endpoints in clinical trials that complement the existing measures. Digital tools can complement existing methods by providing inherently objective measurements, enabling remote patient evaluations. This would dramatically increase data availability for comprehensive assessments, personalized clinical decision-making, and enriched databases for deeper disease understanding. Leveraging advancements in neuroscience, digital technology, and artificial intelligence will drive the development of innovative endpoints for intervention trials, paving the way for more sensitive and accurate evaluations of therapeutic efficacy.

## 8 Conclusion

In this article we reviewed the clinical trials conducted to date on FRDA, with particular attention to the outcome measures used to track changes in patients’ clinical state. Clinical rating scales such as FARS, mFARS, and SARA remain central to trial design, and play a critical role in evaluating symptom severity and disease progression. These scales are frequently complemented by timed-performance measures and PROs, which together provide a broader view of functional status. While each of these tools has certain limitations in sensitivity, objectivity, or specificity, they remain key instruments that have enabled the implementation and interpretation of most trials to date. The widespread use of clinical rating scales has been supported by large-scale natural history studies like EFACTs and FACOMS. These studies have not only confirmed the utility of these instruments but also enhanced our understanding of FRDA progression and informed more targeted and statistically powered trial designs.

At the same time, growing interest in underexplored domains such as cognition and speech highlights important opportunities to expand the scope of outcome assessment. These domains, increasingly recognized as clinically relevant, remain underrepresented in current trials. Consistently including cognitive and speech-related assessments in trial designs could enhance the ability to detect subtle therapeutic effects. In clinical trials, where the expected effects of therapeutic agents are often subtle, even minor improvements can hold meaningful clinical value. As such, evaluation tools must be sufficiently accurate and sensitive to detect these small but meaningful changes (11). Continuous refining of instruments and the development of new tools are therefore of paramount importance (101).

Recent advances in experimental research and digital technologies offer promising avenues to meet these needs. Reaction time-based assessments, precise motor timing tasks, and acoustic speech analysis provide objective, quantitative metrics that can complement traditional measures. The digital implementation of these tools further enhances these approaches, enabling remote and frequent assessments that are both patient-centered and resource-efficient. Notably, recent initiatives, including home based versions of SARA, mobile-health platforms for speech assessment, and wearable and portable devices for passive monitoring, demonstrate the growing feasibility of incorporating these innovations into trials. Instrumented assessments such as the AIM-S, exemplify the potential of technology go generate sensitive and ecologically valid outcome measures.

These efforts are congruent with broader frameworks such as the Ataxia Global Initiative working Group on Digital-Motor Markers, which provides practical recommendations for the integration of such tools, and with regulatory initiatives like the CCTI, which advocates for the modernization of trial design through digital innovation, remote data collection, and patient-centric endpoints.

Looking ahead, continued development, standardization, and validation of these emerging tools, grounded in current experimental knowledge and technological advances, will enable more nuanced and accurate assessments of FRDA across both research and clinical settings. Moreover, the adoption of standardized and objective tools will facilitate data comparability across studies, expand research databases, and accelerate insights into disease mechanisms and treatment responses. By addressing current gaps in evaluation methods and embracing innovation, these advancements have the potential to transform both the conduct of clinical trials and the broader landscape of FRDA research and care.

## Footnotes

^1^ www.frdaguidelines.org

^2^ https://pubmed.ncbi.nlm.nih.gov

^3^ https://ctti-clinicaltrials.org/
