# Supplementary material for: Clinical and cognitive assessment in Friedreich ataxia clinical trials: a review
Source: Front Neurol. 2025 May 22;16:1558493. doi: 10.3389/fneur.2025.1558493 (PMC12142069; doi:10.3389/fneur.2025.1558493)
Supplement: Supplementary file 1 [file Data_Sheet_1.PDF]

Table 1. Summary of the tests used in the published studies addressing cognition in FRDA. Underlined, the cognitive features evaluated, according to the authors. When nothing is underlined, authors did not indicate the features they intended to evaluate. In Bold, tests where significant differences between patients and controls were found. [1] **Tests employing RT and/or MT measures**. [2] **Computerized tests**. (*) Studies which did not involve patient/control comparisons. **7/24 SRT**: 7/24 Spatial Recall Test; **AFT**: Action fluency test; **BDI**: Beck's depression inventory; **COWAT-FAS**: Controlled word association test; **CVLT**: California verbal learning test; **CPT-IP**: Continuous performance test; **DS**: Digit span; **DSB**: Digit span backwards; **DSF**: Digit span forwards; **FRT**: Facial recognition test; **GERT**: Geneva Emotion Recognition Test; **HSCT**: Hayling sentence completion task; **HVLT**: Hopkins verbal learning test; **HVOT**: Hooper Visual Organization Test; **JLOT**: Judgement line orientation test; **MMSE**: Mini-Mental State Examination. **MOCA**: Montreal Cognitive Assessment; **MT**: Movement time; **MWT-B**: Mehrfachwahl-Wortschatz-Intelligenztest. **PASAT**: Paced auditory serial addition test; **RAVLT (-D)**: Rey auditory verbal learning test (-delayed recall); **RCF**: Rey complex figure test; **RCPM**: Raven Coloured Progressive Matrices; **RT**: Reaction time; **SLD**: segment length discrimination task; **SDMT**: Symbol digit modality test; **SPART (-D)**: 10/36 Spatial recall test (-delayed); **SSF/SSB**: Spatial span forward/backward; **TAP**: Test of attentional performance; **TEA:** Test of Everyday Attention; **TMT**: Trail making test; **TMT[R]**: Reitan version of the TMT; **VRT**: Visual recognition test. **WAIS(-R), (-III)**: Wechsler adult intelligence scale (revised), (third version); Wechsler adult intelligence scale (revised); **WCST**: Wisconsin card sorting test; **WISC(-R)**: Wechsler Intelligence Scale for Children (revised); **WMS(-R), (-III)**: Wechsler memory scale (revised), (third version).

#	Source	Clinical rating scales and Cognitive evaluation tests and tools employed
1	(Fehrenbach et al., 1984)	Short-term memory: DS (WAIS).<br>Mathematical abilities: Arithmetic (WAIS).<br>Abstraction, categorization, and linguistic abilities: Similarities (WAIS), Vocabulary (WAIS)<br>Conceptual perception: **Picture Arrangement (WAIS)**<br>Visuoconstructive abilities: **Block Design (WAIS)**, **Three-dimensional mental folding**.<br>Intelligence: RSPM<br>Abstraction, categorization, shift of behavioural strategies: WCST<br>Learning and memory: Sequential Concept Formation.
2	(R. P. Hart et al., 1985)	*Clinical evaluation: Inherited Ataxias Progression Scale.*<br><br>Information processing speed**: Short-term memory scanning procedure (information processing speed)[1,2]**<br>Vocabulary and Information (WAIS-R)<br>Auditory vigilance test.<br>Logical memory (WMS)<br>Categories test<br>Facial recognition memory test.<br>Verbal learning task<br>Verbal subtests (WAIS-R)<br>Token test<br>Visual confrontation naming (Boston diagnostic aphasia exam)<br>Measure of stereognosis and praxis<br>Bender Visual-motor gestalt designs<br>Picture absurdities (Stanford-Binet test of intelligence)
3	(R. P. Hart et al., 1986)	*Clinical evaluation: Inherited Ataxias Progression Scale.*<br><br>Information processing speed**: Short-term memory scanning procedure (information processing speed)[1,2]**<br>Seashore Rhythm Test<br>Category Test<br>Visual confrontation naming (Boston Diagnostic Aphasia Exam)

		Token test<br>Benton visual retention test-multiple choice<br>Sentence repetition from the Benton multilingual aphasia examination.<br>Verbal learning task employing a 12-word list and the selective reminding procedure (Buschke 1973)
4	(Botez-Marquard & Botez, 1993)	*Clinical evaluation: routine neurological examination to rule Parkinsonian rigidity, Hamilton scale for depression as exclusion criterium.*<br><br>Information processing speed (IPS): **Simple Visual RT**[1], **Simple Auditory RT**[1], **Visual movement time (VMT)**[1], **Auditory movement time (AMT)**[1].<br>General intelligence: **RSPM series A, B, C, D and E**.<br>Verbal and non-verbal acquired abilities: DSF/DSB (Ottawa-Weschler), Similarities (Ottawa-Wechsler), Picture arrangement (Ottawa-Wechsler), **Block design (Ottawa-Wechsler)**.<br>Learning and memory: RAVLT.<br>Perceptual organization, programming and planning ability: **Rey complex figure (copy)**.<br>Visuospatial analysis in the absence of movement: HVOT.
5	(White et al., 2000)	*Clinical evaluation: SCL-90, Hamilton depression scale, Semi-structured interviewing relevant for DSM-IV criteria of major depressive disorders.*<br><br>Information processing speed: **reaction time and movement time in a visual RT task**[1], **Stroop**.<br>Executive functions: WCST.<br>Procedural learning: Tower of Hanoi.<br>Verbal fluency: Phonetic verbal fluency (FAS), Semantic verbal fluency (animals),<br>Intelligence: Picture arrangement, Similarities, **Block design** (Ottawa-Wechsler)
6	(Wollmann et al., 2002)	*Clinical evaluation: Clinical Rating Scale (Appollonio et al., 1993)*<br><br>General cognition: MMSE.<br>Attention: Auditory A's Test, Stroop; **DS (WMS-R)**, VS (WMS-R).<br>Reaction Time and Movement Time: **Reaction Unit of the PC-Vienna System (RT and MT)**[1,2].<br>Executive functions: WCST; Similarities Subtest (WAIS-III); **COWAT-FAS**, **Animal Test**.<br>Memory and learning: Recognition version VRT, form C, form F; Modified version of the 7/24 SRT; CVLT, form I; **Logical Memory subtest (WMS-R)**.<br>Visuoperceptive, visuospatial, and visuoconstructive abilities: Computerized version of the JLOT[2]; **computerized version of the FRT**[2]; abbreviated HVOT, **modified Block Design (WAIS-III)**
7	(Mantovan et al., 2006)	Intelligence: WAIS-R, WISC-R<br>Language: **Boston Naming Test**, **Verbal Fluency**.<br>Memory: **DSF/DSB (WAIS-R)**, **WMS**.<br>Calculation: Arithmetic facts (eight single-digit operations), Procedures (six written operations). Attention/information processing/planning: **Attentive Matrices**, **TMT-A, TMT-B**, **Stroop (computerized version of Navon's Test)**[1,2], **Simple visual Reaction Time (Go/NoGo)**[1,2], **Tower of London**.<br>Motor performance: **Computerized finger tapping task**[1,2].<br>Implicit learning: **ad hoc implicit learning task**[1,2]
8	(de Nóbrega et al., 2007)	*Clinical evaluation: Rankin Incapacity Ataxia Scale, Nobile-Orazio Ataxia Scale*<br><br>General cognition: MMSE.<br>General Intelligence: Information Subtest (WAIS-III)<br>Phonemic verbal fluency: **COWAT-FAS**,<br>Semantic verbal fluency: Animal test.<br>Action fluency: **AFT**.<br>Reaction time and Movement time: **Reaction Unit of the PC-Vienna System (RT and MT)**[1,2]<br>Oral agility: **articulatory task of the Boston Diagnostic Aphasia Examination**.
9	(Corben, Delatycki, et al., 2010)	*Clinical evaluation: FARS, BDI*<br><br>General cognition: MMSE.

		Motor reprogramming: **ad hoc designed, computerized reprogramming task measuring down time (RT) and MT as measures of movement preparation and execution, respectively[1,2]**.<br>Planning and inhibitory processes: TMT A and B, Stroop
10	(Fielding et al., 2010)	*Clinical evaluation: FARS, SLCLC*<br><br>Higher order (cognitive) control of volitional eye movements: **Ad hoc designed, computerized task[1,2].**
11	(Hocking et al., 2010)	*Clinical evaluation: FARS, SLCLC*<br><br>Cognitive control of ocular motor fixation in relation to visual attention orienting: **Ad hoc designed, computerized task[1,2].**
12	(Corben, Delatycki, et al., 2011)	*Clinical evaluation: FARS, BDI*<br><br>Utilisation of advance motor information: **ad hoc designed, computerized task[1,2].**<br>Planning and inhibitory processes: TMT A and B, Stroop
13	(Corben, Georgiou-Karistianis, et al., 2011)	*Clinical evaluation: FARS, National Adult Reading Test (NART), BDI*<br><br>Planning and online control of movement: **ad hoc designed, computerized motor task incorporating Fitts' Law[1,2].**<br>Planning and inhibitory processes**: TMT-A, TMT-B**, Stroop
14	(Corben, Akhlaghi, et al., 2011)	*Clinical evaluation: FARS, National Adult Reading Test (NART), BDI*<br><br>Planning and inhibitory processes**: TMT-A, TMT-B**, Stroop<br>Response inhibition: **Simple RT (SRT)[1,2]**, Choice RT (CRT)[1,2], **Simon Task[1,2]**.
15	(Klopper et al., 2011)	*Clinical evaluation: FARS, National Adult Reading Test (NART)*<br><br>Sustained volitional attention: Elevator Counting (TEA), **The Lottery subtest (TEA)**<br>Working memory and sustained volitional attention: **Elevator Counting with Distraction (TEA)**<br>Working memory with sustained volitional attention contribution: **Visual Elevator (TEA)**<br>Working memory with sustained volitional attention contribution: **Elevator counting with Reversal (TEA)**
16	(Georgiou-Karistianis et al., 2012)	*Clinical evaluation: FARS, National Adult Reading Test (NART), BDI*<br><br>Executive function: **Computerized Simon[1,2]**.
17	(Nieto et al., 2012)	*Clinical evaluation: Rankin Incapacity Ataxia Scale, Nobile-Orazio Ataxia Scale, Clinical Rating Scale (Appollonio et al., 1993)*<br><br>Global screening**:** Mini-Mental State Examination (MMSE), Information Subtest (WAIS-III), BDI.<br>Information speed processing, motor and coordination processing: **Simple reaction time, simple motor time (Pc-Vienna System), choice decision time, choice motor time (Pc-Vienna System)[1,2]**.<br>Sustained attention: computerized version of CPT-IP[2].<br>Selective attention: Stroop.<br>Working memory: DSF/DSB, (WMS-III), SSF/SSB (WMS-III).<br>Executive functions**:** WCST, **Similarities Subtest (WAIS-III)**, Verbal fluency **(FAS, Animals, Actions)**.<br>Verbal memory**: Logical Memory (WMS-III)**, **CVLT**.<br>Visual memory: 10/36 SRT<br>Visuoperceptive, visuospatial, and visuoconstructive abilities**:** JLOT, **FRT**, Minnesota paper form board test, **Block Design (WAIS-III)**<br>Language**:** specifically designed tasks including computerized noun naming[2], **action naming[2]**, and anaphora comprehension[2]
18	(Nieto et al., 2013)	*Clinical evaluation: Rankin Incapacity Ataxia Scale, Nobile-Orazio Ataxia Scale, Clinical Rating Scale (Appollonio et al., 1993)*<br><br>Global screening**:** Mini-Mental State Examination (MMSE), Information Subtest (WAIS-III), BDI.<br>Information speed processing, motor and coordination processing: **Simple reaction time**, **simple motor time (Pc-Vienna System)**, **choice decision time**, **choice motor time (Pc-Vienna System)[1,2]**.<br>Sustained attention: computerized version of CPT-IP[2].<br>Selective attention: Stroop.

		Working memory: DSF/DSB, (WMS-III), SSF/SSB (WMS-III).<br>Executive functions**:** WCST, **Similarities Subtest (WAIS-III)**, Verbal fluency **(FAS**, **Animals**, **Actions)**.<br>Verbal memory**:** Logical Memory (WMS-III), CVLT.<br>Visual memory: 10/36 SRT<br>Visuoperceptive, visuospatial, and visuoconstructive abilities**:** JLOT, **FRT**, Minnesota paper form board test, **Block Design (WAIS-III)**<br>Language**:** specifically designed tasks including computerized noun naming[2], computerized action naming[2], Anaphora comprehension[2]
19	(Akhlaghi et al., 2014)	*Clinical evaluation: FARS, BDI*<br><br>Executive function: **Computerized Simon[1,2].**
20	(Hocking et al., 2014)	*Clinical evaluation: FARS, SLCLC*<br><br>Cognition: **Ad hoc designed saccadic oddball task** aimed at examining the ability to inhibit a prepotent response and reprogram a new response to correspond with changes in either the amplitude or direction of an oddball target.[2]
21	(Nachbauer et al., 2014)	*Clinical evaluation*: *SARA, 8MWT, 9HPT, PATA, International Reading Speed test, Vocabulary test, ADL.*<br><br>Lexical search and monitoring: **Semantic verbal fluency (animals/minute)**, **Phonetic verbal fluency (s-words/minute)**.<br>Problem solving, planning and working memory: Tower of London[2].<br>Selective attention and inhibition of interference: **Stroop**.<br>Working memory: DSF/DSB (WAIS-R).<br>Attention, reaction to visual stimulus and alertness: **Intrinsic Alertness task (TAP)[1,2.]**<br>Attention, reaction to visual stimulus after warning (tone): **Alertness after warning (TAP)[1,2]**.<br>Attention, parallel processing of visual (geometrical symbols) and auditory stimuli (high and low tones): **Divided attention (TAP)[1,2]**.<br>Visual perception and global processing: Incomplete letters (VOSP)<br>Spatial perception: Position discrimination (VOSP).<br>Learning and memory**:** Verbal learning and retention memory test.
22	(Dogan et al., 2016)	*Clinical evaluation: SARA, INAS, SCAFI (8MWT, 9HPT, PATA)*<br><br>General cognitive impairment: MOCA.<br>Premorbid intelligence level: Multiple choice vocabulary test (MWT-B).<br>Verbal learning and memory: CVLT.<br>Attention and working memory: DSF/DSB (WMS), **PASAT**.<br>Selective attention and cognitive control: Stroop.<br>Verbal fluency: **Phonemic (A, F)**, **Semantic (Food)**.<br>Social cognition: **Faux-Pas-Recognition test**.
23	(Harding et al., 2016)	*Clinical evaluation*: *FARS*<br><br>Executive, memory, and attentional processes: n-back working memory task[1,2]
24	(Corben et al., 2017)	*Clinical evaluation*: FARS<br><br>Inhibitory processes and cognitive flexibility: **HSCT**, TMT(B-A), Stroop.
25	(Cocozza et al., 2018)	*Clinical evaluation: SARA*<br><br>Global assessment: **MOCA**.<br>Language: Naming nouns, Pointing names.<br>Intelligence: RCPM.<br>Executive functions: **SDMT**, Attentional Matrices, **TMT-A, TMT-B**, **Stroop**, Weigl's Sorting Test, **Phonetic fluency, Semantic Fluency**.<br>Memory: DS, **SPART**, RAVLT.<br>Visuoperception and visuospatial functions: **SLD**, **Mental rotation**.
26	(Costabile et al., 2018)	*Clinical evaluation: SARA*<br><br>Global functioning: **MOCA**<br>Verbal working memory: DS.<br>Spatial short and long-term memory: **SPART**, SPART-D<br>Verbal short and long-term memory: RAVLT/RAVLT-D<br>Executive functions: **TMT-A**, **TMT-B**, **TMT B-A**, **Stroop**, **Semantic fluency**, **Phonetic fluency**.

		Faces recognition: Physiognomic decision test.<br>Emotion: **GERT**
27	(Nieto et al., 2018)*	*Clinical evaluation: SARA, BDI-II*<br><br>Information processing speed: Correlation between depressive symptoms (BDI) and information processing speed. **Depressive symptoms predict decision reaction times, as measured using the Choice Reaction Time task using the Reaction Unit/Vienna System** [1,2].
28	(Saccà et al., 2018)*	*Clinical evaluation: SARA, PATA, 9HPT*<br><br>**Attention matrices**<br>**TMT-A**, **TMT-B**<br>**SDMT**<br>**Phonemic fluency**<br>**Semantic fluency**
29	(Sayah et al., 2018)*	*Clinical evaluation: SARA*<br><br>Global cognition: RCPM.<br>Semantic capacities: **Mill-Hill part B**.<br>Verbal learning and memory: HVLT<br>Attentional skills: **PASAT**.<br>Focused attention: **Stroop**.<br>Mental flexibility: P-fluency test<br>Social cognition and emotional recognition: mini–Social Cognitive and Emotional Assessment (SEA), Faux-Pas Recognition test.<br>Emotional recognition: Ekman facial expressions.<br>Depression: BDI
30	(Vavla et al., 2018)	*Clinical evaluation: SARA, ICARS, FARS.*<br><br>General cognitive assessment: WISC-III, WAIS-R
31	(Cocozza et al., 2020)	*Clinical evaluation: SARA, 9HPT to correct TMT, PATA to correct SDMT*<br><br>Memory: **SPART**, **SPART-D**.<br>Visuoperception and visuospatial functions: **SLD**.<br>Executive functions: **SDMT**, Stroop, **TMT-A**, **TMT-B**
32	(Naeije et al., 2020b)	*Clinical evaluation: SARA*<br><br>Language, executive function, visuo-spatial skills: **CCAS-S**
33	(Shishegar et al., 2020)	*Clinical evaluation: FARS for evaluating clinical severity*<br><br>Language: **HSCT**<br>Executive function**: Stroop**, **TMT-A**, **TMT-B**, **TMT B-A**<br>Working memory: **DSF**, **DSB**, N-back working memory task[1,2]
34	(Hernández-Torres et al., 2021) *	*Clinical evaluation: Rankin Disability Scale, Clinical Rating Scale (CRS)*<br><br>* Longitudinal study, progression of disease over an 8-year average.<br>Global screening: MMSE, Information subtest (WAIS-III), BDI.<br>Simple and choice reaction times: **Simple Decision Time**[1], **Simple motor time (Pc-Vienna System)**, **Choice Decision Time**[1,2], **Choice motor time (Pc-Vienna System)**[1,2].<br>Sustained attention: computerized CPT[2].<br>Selective attention: **Stroop**.<br>Working memory: DSF, DSB, SSF, SSB (WMS-III)<br>Executive functions: WCST, Similarities (WAIS-III), Verbal fluency: phonemic (FAS), semantic (**Animals)**, Actions.<br>Verbal memory: CVLT, logical memory subtest (WMS-III).<br>Visual memory: SPRT.<br>Visuoperceptive and visuospatial skills: FRT, JLOT.<br>Visuoconstructive skills: **Block design (WAIS-III)**.<br>Language: noun and action naming, anaphora task.
35	Thieme et al., 2022	*Clinical evaluation: SARA, INAS*<br>Language, executive function, visuo-spatial skills: **CCAS-S**
36	Destrebecq et al., 2023	*Clinical evaluation: SARA*<br><br>Language, executive function, visuo-spatial skills: **CCAS-S**

37	Corben et al., 2024	*Clinical evaluation: FARS, SARA (speech item)*
		Language, executive function, visuo-spatial skills: **CCAS-S.**
