# Supplementary material for: Clinical and cognitive assessment in Friedreich ataxia clinical trials: a review
Source: Front Neurol. 2025 May 22;16:1558493. doi: 10.3389/fneur.2025.1558493 (PMC12142069; doi:10.3389/fneur.2025.1558493)
Supplement: Supplementary file 2 [file Data_Sheet_2.PDF]

Table 2. Summary of the **interventional** FRDA clinical trials registered in the websites ClinicalTrials.gov (marked as US- in the "Clinical Trial" column") and clinicaltrialsregister.eu (marked as EU-), indicating the primary and secondary (*Italic*) outcome measures employed. In grey, the studies not including any neurological, motor, or cognitive assessment. Please, note that entries corresponding to different phases or extensions of the same clinical trial have the same index number in the first column. Also, note that in some cases the associated publications cannot be found in the websites, but are published elsewhere. **1MWD:** 1-minute walking distance; **6MWT:** Six-minute walk test; **9HPT:** 9-Hole Peg Test; **ADL:** activities of daily living; **AEs:** adverse events; **BNP:** B-type natriuretic peptide; **CCFS:** Composite Cerebellar Functional Severity; **CGI-C:** Clinician's global impression-Change scale; **CGI-I:** Clinician Global Impression-Improvement; **CGI-S:** Clinical Global Impression score; **CPET:** cardiopulmonary exercise test; **CPK:** creatine phosphokinase; ***CrCEST*** **:** Creatine chemical exchange saturation transfer; **EchoCG:** echocardiogram; **EKG:** electrocardiogram; **F8WT:** Figure of 8 Walk Test; **ETDRS:** Early treatment diabetic retinopathy study; **FAIS:** Friedreich Ataxia Impact Scale; **GICS:** Global Impression of Clinical Severity. **HSCT:** (Junior) Hayling Sentence Completion Test; **HADS:** Hospital anxiety and depression scale; **IADL:** instrumental activities of daily living scale; **HbA1C:** haemoglobin A1C test; **ICARS:** International Cooperative Ataxia Rating Scale; **IVGTT:** intravenous glucose tolerance test; **LCLA:** Low-contrast Letter Acuity; **LCSLC:** Low-contrast Sloan Letter Chart; **LiSN-S:** Spatial auditory information; **(m)FARS:** (modified) Friedreich Ataxia Rating Scale; **MRI:** magnetic resonance image; **(m)RNA:** (mitochondrial) ribonucleic acid; **N:** Number of participants; **PGI-I:** Patient Global Impression-Improvement; **PGI-S:** Patient Global Impression score; SAEs: Severe adverse events; **SARA:** Scale for the Assessment and Rating of Ataxia; **SF-36:** 36-item short form survey for quality of life; **TEAEs:** treatment emergent adverse events; **T25FW:** timed 25-foot walk; **TfR1:** Transferrin receptor protein 1; **WHO-DAS 2.0:** World Health Organization Disability Assessment Schedule 2.0.

#	Clinical trial	Intervention	Phase	Date	N	Primary and *secondary* outcome measures	Results and related publications
1	**US- Effect of iron-chelating therapy in Friedreich Ataxia. Study Phase I/II FRDA.**	Iron chelating intervention	1-2	March 2005-March 2008	15	- Assessment of iron overload at TP and month 2 by imagery.<br>*- Clinical (ICARS) and biological parameter follow-up.*<br>*- Plasma Iron, ferritin, transferrin and liver enzymes.*	**- Significant removal of labile iron accumulated in the dentate nucleus after 6-month treatment.**<br>**- Significant improvements in ICARS scores after 6-month treatment.**<br>**- Significant improvement in the Perdue Pegboard test on three randomly selected participants.**<br><br>***Published results (Boddaert et al., 2007)***
2	US- Phase I clinical trial to establish the maximum tolerated dose of Idebenone in children, adolescents, and adults with Friedreich Ataxia.	Idebenone	1	May 2001-April 2006	100	--	See next entry
2	US- Phase 1B clinical trial to establish the safety and tolerability of a multiple-dose regimen of Idebenone administered to patients with FRDA.	Idebenone	1	February 2004-April 2006	16	--	See next entry
2	**US- A six month double-blind, placebo-controlled Phase 2 clinical trial to determine the safety and efficacy of Idebenone administered to patients with Friedreich Ataxia.**	Idebenone	2	September 2005-December 2007	51	- To examine the change in the level of oxidative stress by measuring the oxidative marker 8-hydroxy-2-deoxyguanosine (8-OHdG) from baseline and after 6 months of treatment with placebo or varying doses of Idebenone.<br>*- To evaluate the safety and tolerability of Idebenone.*<br>*- To explore the effects of Idebenone on cardiac parameters.*<br>*- To explore the effects of Idebenone on neurological function (ICARS, FARS, ADL)*<br>*- To explore the effects of Idebenone on patients' quality of life.*<br>*- To explore the effects of Idebenone on functional capacity.*<br>*- To evaluate metabolic alterations, gene expression changes and markers of mitochondrial DNA damage.*	- No significant changes from baseline in 8-hydroxy-2-deoxyguanosine<br>- No significant effects of treatment among the groups (placebo, low dose, intermediate dose, high dose) in the degree of change from baseline in ICARS, FARS, or ADL.<br>**- When excluding participants who required a wheelchair, significant differences were observed between intermediate and high dose groups and the placebo group in ICARS total score, but not in FARS or ADL.**<br><br>***Published results (Di Prospero et al., 2007)***
3	US- EU- A Phase III double-blind, randomized, placebo-controlled study of the efficacy, safety and tolerability of Idebenone in the treatment of Friedreich Ataxia patients	Idebenone<br><br>Placebo	3	April 2006-January 2010	232	- Absolute change in ICARS scores from baseline to week 52.<br>*- Absolute change in FARS from baseline to week 52.*<br>*- Proportion of patients improving on ICARS by a clinically relevant margin.*<br>*- Proportion of patients improving on left ventricular peak systolic strain rate or showing a reduction in left ventricular mass index with no worsening in strain rate.*	- No significant changes in ICARS.<br>*- No significant changes in FARS.*<br>*- No significant changes in any other variable.*

						- Change in peak systolic strain rate from baseline to week 52.<br>*- Change in peak workload baseline to week 52.*	
3	US- EU- A phase III open-label, single-group extension study to obtain long-term safety and tolerability data of Idebenone in the treatment of Friedreich Ataxia patients.	Idebenone	3	June 2007- June 2012	200	- Measures of safety and tolerability: nature and frequency of AEs.<br>- Absolute change in the ICARS.<br>*- Measures of safety and tolerability: physical examinations and vital signs.*<br>*- Measures of safety and tolerability: EKG.*<br>*- Measures of safety and tolerability: haematological and biochemical laboratory parameters.*	- No significant changes in ICARS.
3	**US- EU- A Phase IIIb double-blind, randomized, placebo-controlled study of patient reported outcomes in Friedreich's Ataxia patients after withdrawal from treatment with Idebenone (PROTI Study)**	Idebenone<br><br>Placebo	3	April 2011- July 2012	29	- Patient assessment of treatment assignment.<br>*- Comparison of the percentage of participants randomized to Idebenone and placebo who withdrew early due to recurrence or worsening of FRDA symptoms.*	- Patients were not able to determine their treatment assignment.<br>- No significant differences in Modified Fatigue Impact Scale (MFIS).<br>- No significant differences in 9HPT.<br>- No significant differences in CGI-C<br>**- Significant effect on the AIDS speech capability test.**<br>- No significant differences in ICARS overall.<br>**- Significant effect on ICARS when considering ambulatory patients only.**<br><br>***Published results (Cook et al., 2019)***
4	**EU- Eine offene, nicht randomisierte, einarmige Pilotstudie zur Beurteilung der Wirksamkeit von Erythropoetin bei Friedreich Ataxie.**	Erythropoietin	2	June 2006- November 2007	12	- Frataxin levels.<br>- Urine 8-OHdG for oxidative stress.<br>- FARS.<br>- SARA.<br>- SF-36	**- Significant effects on Frataxin levels.**<br>**- Significant effects on 8-OHdG**<br>**- Significant effects on FARS score.**<br>**- Significant effects on SARA score.**<br>**- Significant effects on SF-36 score.**<br><br>***Published results (Boesch et al., 2007, 2008)***
4	**EU-Untersuchung der Dosis-Wirk-Beziehung unterschiedlicher Erythropoetin-Dosen auf Frataxin bei Friedreich Ataxie**	Erythropoietin<br><br>Placebo	2	December 2007- December 2011	5	- Monthly Frataxin Measurements.<br>- Clinical rating scales.<br>- Mitochondrial function measures.	**- Significant increase of frataxin levels.**<br>**- Significant increase of serum erythropoietin levels.**<br>**- Significant decrease of ferritin.**<br>**- Significant improvement of mitochondrial function.**<br>- No significant effects (benefits) on SARA scores.<br><br>***Published results (Nachbauer, Hering, et al., 2011)***
4	**EU- Effects of recombinant human Erythropoietin on circulating and intramuscular endothelial progenitor cells, neovascularization and oxidative metabolism of skeletal muscle in Friedreich Ataxia.**	Erythropoietin	2	February 2009- August 2012	7	- Inorganic phosphate (resting state)<br>- Relative and absolute changes in phosphocreatine (PCr), inorganic phosphate (Pi) and adenosine triphosphate (ATP)<br>- SARA.	**- Changes in PCr of the recovery phase correlate with SARA score.**<br>- Changes in Pi do not correlate with SARA scores.<br>**- SARA scores correlate with several biochemical parameters.**<br><br>***Published results (Nachbauer et al., 2013)***
5	**EU- Randomized placebo-controlled**	Erythropoietin	2	October 2007-	18	- Increased level of frataxin protein in peripheral lymphocytes.	- For results related to this trial, see Table 4 (Mariotti et al., 2012)

	**double-blind trial to assess safety and efficacy of erythropoietin in adult patients with Friedreich Ataxia (a pilot study)**	Placebo				- ICARS.<br>- SF-36.<br>- Echocardiogram.<br>- Skin biopsy: observation of sensory nerve fibre regeneration.	
6	**EU- Efectos del tratamiento con Deferiprone sobre la hipertrofia miocárdica y los parámetros de función cardiaca en pacientes afectos de Ataxia de Friedreich.**	Deferiprone<br><br>Idebenone	4	December 2007-	12	- Measures of cardiac hypertrophy and cardiac function parameters.<br>- Clinical rating scales.<br>- MR to measure the effect of deferiprone on the iron deposits in the brain.	- For results related to this trial, see Table 4 (Arpa et al., 2014)
7	**US- A Phase III double-blind, randomized, placebo-controlled study of the efficacy, safety and tolerability of Idebenone in the treatment of Friedreich Ataxia patients.**	Idebenone<br><br>Placebo	3	December 2007-April 2009	70	- ICARS.<br>*- FARS.*<br>*- ADL of FARS.*<br>*- FACT (derived from T25FW and 9HPT).*<br>*- LCLA*	- No significant effects on ICARS.<br>*- No significant effects on FARS.*<br>*- No significant effects on ADL.*<br>*- No significant effects on FACT.*<br><br>***Published results (Lynch et al., 2010)***
7	**US- A Phase III open-label, single group extension study of the safety and tolerability of Idebenone in the treatment of Friedreich Ataxia patients**	Idebenone	3	July 2008-May 2010	68	- Change in ICARS.<br>*- FARS.*<br>*- Nature and frequency of adverse events.*	- No effects on ICARS total score after 12 or 18 months.<br>**- Significant effects on ICARS subscales after 12 months:**<br>• **Improvement on speech subscore.**<br>• **Worsening on posture and stance subscore.**<br>**- Significant effects on ICARS after 18 months:**<br>• **Improvement on eye subscore.**<br>• **Improvement on speech subscore.**<br>• **Improvement on upper limb ataxia subscore.**<br>• **Improvement on lower limb ataxia subscore.**<br>• **Worsening on posture and stance subscore.**<br><br>***- Significant effects on FARS after 12 months:***<br>• ***Worsening on total score.***<br>• ***Worsening on PNS subscore.***<br>• ***Worsening on stability and gait subscore.***<br>• ***Improvement on speech subscore.***<br>***- Significant effects on FARS after 18 months:***<br>• ***Worsening on total score.***<br>• ***Worsening on PNS subscore.***<br>• ***Worsening on stability and gait subscore.***<br>• ***Improvement on speech subscore.***<br>• ***Improvement on lower limb coordination.***<br><br>***Published results (Meier et al., 2012)***

8	**US- Single-center, open-label, sequential trial to test the efficacy, safety and tolerability of Epoetin Alfa in patients with Friedreich Ataxia**	Epoetin Alfa	2	February 2008-June 2009	10	- Frataxin level in PBMCs from patients at different timing from a single Epoetin alfa administration.<br>*- Echocardiography: strain and strain rate after EPO administration at the highest study dose.*<br>*- Safety laboratory parameters, adverse events and tolerability.*<br>*- ICARS.*	**- Significant increase in frataxin 3 months after the first dose and up to 6 months after the second dose.**<br>*- No changes in echocardiographic measures.*<br>*- No effects on ICARS.*<br><br>***Published results (Saccà et al., 2011)***.
8	**US- EU- A double-blind, randomized, placebo-controlled, clinical trial to test the efficacy of Epoetin Alfa on physical performance of Friedreich Ataxia patients.**	Epoetin alfa	2	January 2013-June 2015	56	- Peak oxygen uptake (VO2 max) at the CPET.<br>*- Secondary outcome variables at the CPET.*<br>*- Frataxin levels in peripheral blood mononuclear cells.*<br>*- EKG.*<br>*- Vascular reactivity.*<br>*- Neurological progression measured with SARA and 9HPT.*<br>*- Quality of life assessed with the EQ-5D, ADL, and IADL scales.*<br>*- Safety and tolerability assessed by recording all serious and non-serious AEs.*	- No significant effects on VO2 max.<br>- No significant effects on secondary outcome variables at the CPET.<br>- No significant effects on Frataxin levels.<br>- No significant effects on SARA scores.<br>**- Significant amelioration in 9HPT in the treatment group.**<br>- No significant effect in EQ-5D, ADL, and IADL scales.<br><br>***Published results (Saccà et al., 2016)***
9	**US- EU- A six month double-blind, randomized, placebo-controlled study investigating the safety and tolerability of Deferiprone in patients with Friedreich Ataxia.**	Deferiprone<br><br>Placebo	1-2	April 2008-July 2009	80	- Patient's tolerance of treatment will be determined, as assessed by the occurrence of adverse events.<br>*- 9HPT*<br>*- T25FW.*<br>*- LCLA.*<br>*- ICARS.*<br>*- FARS.*	- Drug well tolerated at 20 mg/kg/day, more adverse events at 40 mg/kg/day, treatment discontinued at 60 mg/kg/day due to worsening of ataxia in 2 patients.<br>*- No significant effects on ICARS and FARS scores with 20 mg/kg/day.*<br>***- Significant worsening of ICARS and FARS scores with 40 mg/kg/day.***<br>*- No significant effects on 9HPT.*<br>*- No significant effects on T25FW.*<br>*- No significant effects on LCLA.*<br><br>***Published results (Pandolfo et al., 2014)***
9	US- EU- An open-label, single treatment, safety and efficacy, long-term study of Deferiprone in subjects with Friedreich Ataxia	Deferiprone	2	June 2009-March 2011	36	- Patient's long-term tolerance of treatment assessed by the occurrence TEAEs.<br>*- 9HPT*<br>*- T25FW*<br>*- LCLA*<br>*- ICARS*<br>*- FARS.*	- No further results found (see previous entry).
10	US- EU- Efficacy of EGb761 120mg BID versus placebo in patients suffering from Friedreich Ataxia. A 3-month, Phase II, randomized, double blind, placebo controlled, parallel group clinical study.	EGb 761<br><br>Placebo	2	June 2008-October 2011	22	- Creatine rephosphorylation rate post exercise.<br>*- Time to peak perfusion.*<br>*- Perfusion-time integral during the first 9 minutes post exercise.*<br>*- Muscle trophicity: maximum cross section of muscle.*<br>*- Developed force during the exercise bout.*<br>*- Normalized work developed during the exercise.*<br>*- Metabolism efficacy index.*<br>*- ICARS total score.*<br>*- ICARS posture and gait disturbance score.*<br>*- ICARS kinetic function score.*<br>*- ICARS speech disorders score.*<br>*- T25FWT.*	- No significant effects on Creatine rephosphorylation rate post exercise.<br>*- No significant effects on time to peak perfusion.*<br>*- No significant effects on perfusion-time integral during the first 9 minutes post exercise.*<br>*- No significant effects on muscle trophicity: maximum cross section of muscle.*<br>*- No significant effects on developed force during the exercise bout.*<br>*- No significant effects on normalized work developed during the exercise.*<br>*- No significant effects on metabolism efficacy index.*<br>*- No significant effects on ICARS total score or any ICARS subscale.*

						- 9HPT (dominant and nondominant hands).<br>*- Choice reaction time test – Reaction time.*<br>*- Choice reaction time test – Movement time.*<br>*- Visual assessment scale of global impression – patient.*<br>*- Visual assessment scale of global impression – parents.*<br>*- Visual assessment scale of global impression – investigator.*	***- Significant effects on T25FWT.***<br>*- No significant effects on 9HPT.*<br>*- No significant effects on Choice reaction time test - Reaction Time.*<br>*- No significant effects on Choice reaction time test – Movement Time.*<br>*- No significant effects in any visual assessment scale of global impression.*
11	US- Effect of Pioglitazone administered to patients with FRDA: proof of concept.<br>EU- Étude de l'effet de la Pioglitazone dans l'ataxie de Friedreich: étude « preuve de concept ».	Pioglitazone<br><br>Placebo	3	December 2008-<br>March 2013	40	- ICARS.<br>- Tolerance of Pioglitazone.<br>- Efficacy of Pioglitazone on neurological function.<br>- Efficacy of Pioglitazone on functional handicap and quality of life.<br>- Effect of Pioglitazone on cardiac parameters.	- No posted or published results found.
12	US- Pilot study of Varenicline (Chantix®) in the treatment of Friedreich Ataxia.	Varenicline<br><br>Placebo	2-3	May 2009-<br>May 2010	28	- FARS.	- No posted or published results found.
13	**US- EU- Randomized, double blind, placebo-controlled study of Lu AA24493 in patients with Friedreich Ataxia to evaluate safety and tolerability and to explore efficacy**	Lu AA24493<br><br>Placebo	2	October 2009-<br>April 2011	36	- To evaluate the safety and tolerability of 2 weeks treatment with Lu AA24493 in patients with FRDA.<br>*- To explore biomarkers of efficacy, including Frataxin, 8-OHdG & peroxides.*<br>*- To explore efficacy by neurological assessment (SARA, FARS)*<br>*- To explore efficacy by the Clinical Global Impression scales.*<br>*- To explore population pharmacokinetic parameters of Lu AA24493.*<br>*- To evaluate the immunogenicity of Lu AA24493.*	- No significant effects on biomarkers.<br>- No significant effects on neurological rating scales scores.<br><br>***Published results (Boesch et al., 2014)***
14	**US- A Phase 2a, double-blind, randomized, placebo-controlled, 28 day, three-arm, parallel group study of A0001 in the treatment of subjects with Friedreich Ataxia.**	Alpha-tocopherolquinone (A0001)<br><br>Placebo	2	December 2009-March 2011	42	- Change in disposition index (DI) of glucose regulation, determined by frequent sampling IV glucose tolerance test.<br>*- Sensitivity index calculated from IVGTT.*<br>*- Glucose effectiveness calculated from IVGTT.*<br>*- AIRg for glucose and insulin during IVGTT.*<br>*- Fasting glucose, insulin and lactate.*<br>*- HbA1C test.*<br>*- Plasma 1,5-anhydroglucitol.*<br>*- Specific activity of complex 1 in whole blood.*<br>*- T25WT.*<br>*- FARS.*<br>*- 9HPT.*<br>*- Vision low contrast letter acuity test.*<br>*- Global impression of clinical severity.*<br>*- Modified fatigue impact scale.*<br>*- Activities of daily living.*<br>*- SF-36.*	- No effects on the DI.<br>*- Improvement on FARS total score.*<br>*- Improvement on lower limb FARS subscore.*<br>*- No significant effects on T25WT.*<br>*- No significant effects on 9HPT.*<br>*- Significant effects on GICS.*<br><br>***Published results (Lynch et al., 2012)***

15	US- Emergency use protocol for EPI-743 in acutely Ill patients with inherited mitochondrial respiratory chain disease within 90 days of end-of-life care.	EPI-743	2	February 2010-December 2022	94	- Number of participants experiencing AEs.<br>- Change from baseline in neurological function, as determined by standard neurological examination at week 13.<br>- Change from baseline in Newcastle Pediatric Mitochondrial Disease Score (NPMDS) at week 13.	
15	**US- Safety and efficacy of EPI-743 on visual function in patients with Friedreich Ataxia.**	EPI-743<br>Placebo	2	December 2012-February 2016	60	- Visual function: low contrast acuity.<br>*- Colour vision: Roth 28 hue test.*<br>*- Neurologic function: FARS.*<br>*- Neuromuscular function: T25FW.*<br>*- Neuromuscular function: 9HPT.*<br>*- Quality of life: SF-36.*<br>*- Disease biomarkers: blood biomarker levels.*<br>*- Cardiac function: echocardiogram.*<br>*- Safety: number of adverse events.*<br>*- Disease improvement: patient global improvement scale.*<br>*- Visual function: visual field exam.*	- No effects on low-contrast visual acuity.<br>*- No effects on FARS score, T25FW, 9HPT after 6 months.*<br>***- Clinically meaningful improvements in the low-dose EPI-743 group compared to placebo in FARS at 6 months.***<br>***- At 24 months, FARS scores improve 1.8 points for the low-dose group compared with mean worsening of 4.8 points in a matched natural history cohort.***<br><br>***Published results (T. Zesiewicz, Salemi, et al., 2018)***
15	**US- A Phase 2A clinical trial of EPI-743 (Vincerinone ®) on visual function in Friedrich Ataxia patients with point mutations**	EPI-743	2	October 2013-June 2016	4	- Visual function: low contrast acuity.<br>- Safety parameters: clinical and laboratory safety parameters.<br>*- Visual function: Humphrey 30-2 exam. High contrast visual acuity.*<br>*- Physical function: T25FW.*<br>*- Health related quality of life: patient report via rating scale.*<br>*- Activities of daily living: patient report via rating scale.*<br>*- Cardiac indices: echocardiogram.*<br>*- Upper extremity function: 9HPT.*<br>*- Disease biomarkers: Glutathione cycle components.*	**- Total FARS score improvement. Improvement in all subscales, particularly bulbar and upper limb coordination after 6 months.**<br><br>***Published results (Sullivan et al., 2016)***
16	**US- Efficacy of Riluzole in hereditary cerebellar ataxia: a randomized double-blind placebo-controlled trial.**	Riluzole<br><br>Placebo	2-3	April 2010-March 2014	60	- SARA.<br>*- Baropodometric parameters.*<br>*- SF36.*<br>*- Beck Scale for depression.*	**- Significant larger proportion of patients with improved SARA score at month 12.**<br>**- Significant effect on changes in SARA score from baseline to months 3 and 12.**<br>*- No effects on SF-36*<br>*- No effects on Beck Depression Inventory.*<br><br>***Published results (Romano et al., 2015)***
17	**US- An open label clinical pilot study of Resveratrol as treatment for FRDA.**	Resveratrol	1-2	April 2011-December 2012	27	- Lymphocyte Frataxin level.<br>*- Oxidative stress markers.*<br>*- FARS, ICARS, SARA, Friedreich Ataxia Functional Composite.*<br>*- EKG measures.*<br>*- Pharmacokinetic studies of Resveratrol.*	- No effects on frataxin levels.<br>- No improvements in cardiac measures or patient-reported outcome measures.<br>**- The following significant effect were only observed with high doses:**<br>• **On oxidative stress markers.**<br>• **On total FARS score, total ICARS score, FACT Z3 (composite of z scores from 9HPT-1, T25FW-1, and LCLA).**<br>• **On audiologic and speech measures.**

							Published results (Yiu et al., 2015)
17	US- Double-blind, randomised, placebo-controlled 2-period crossover trial of 2g/day of micronized Resveratrol versus placebo. Participants will be randomised in terms of the order in which they receive micronized Resveratrol and placebo.	Resveratrol	2	May 2019-March 2024	25	- Modified FARS.<br>*- 9HPT*<br>*- Berg Balance Scale.*<br>*- Ataxia Instrumented Measure-Spoon.*<br>*- FAIS.*<br>*- Modified fatigue impact scale.*<br>*- Measures of speech (reading a paragraph, produce a prolonged vowel sound for 5", count from 1 to 20, produce a 1-minute monologue on a pre-specified topic.*<br>*- Measures of hearing (LiSN-S).*<br>*- Cardiac parameters measured by echocardiography.*<br>*- Frataxin levels.*<br>*- mRNA levels.*<br>*- Plasma F2-isoprostane levels.*	- No posted or published results found.
18	**EU- Estudio aleatorizado, doble ciego, controlado con placebo, de eficacia, tolerancia y seguridad del tratamiento con el factor trófico IGF-I en la Ataxia de Friedreich.**	Mecasermin (Increlex®): insulin/insulin-like growth factor 1 (IGF-1)<br><br>Placebo	3	February 2012	30	- SARA.<br>- 9HPT.<br>- Click test.<br>- Writing test.<br>- Echocardiogram.<br>- Basal and trimestral frataxin expression.<br>- IGF-1 level changes in blood.	- For results related to this trial, see Table 4 (Sanz-Gallego et al., 2014)
19	**US- EU- Pharmacodynamic studies of a Histone Deacetylase inhibitor in FRDA**	Nicotinamide	2	June 2012-December 2017	40	- Significant upregulation of Frataxin using an antibody dipstick assay.<br>*- SARA.*<br>*- Use of novel highly-sensitive technology to capture clinical deficit (non-interventional part).*<br>*- Correlate functional changes to levels of expression of Frataxin protein and the epigenetic structure of the Frataxin over a 6–9-month period without nicotinamide.*<br>*- Assessment of additional FRDA biomarkers using gene expression profiling.*<br>*- Chromatin immunoprecipitation.*<br>*- Determine the safety and tolerability of nicotinamide in FRDA patients.*	**- In Phase 1, significant dose-response relation for proportional change in frataxin protein concentration from baseline to 8h post-dose, which increased with increasing dose.**<br>**- In Phases 2 and 3, sustained and significant upregulation of frataxin expression, accompanied by a reduction in heterochromatin modifications at the Frataxin locus.**<br>*- No significant effects on SARA, SCAFI, speech dysarthria, and ADL.*<br><br>***Published results (Libri et al., 2014)***
20	EU- Randomized, double-blind, placebo controlled, study of effectiveness, tolerability and safety of therapy with Amantadine in degenerative ataxias.	Amantadine hydrochloride	2	October 2012-	150	- SARA.<br>*- Echocardiography.*<br>*- Neuro-ophthalmology rating.*<br>*- Visual evoked potentials.*<br>*- Otoneurologic review.*<br>*- Brain MRI.*<br>*- Changes in blood levels of IGF-1 and Frataxin.*	- No posted or published results found.
21	US- An objective double-blind evaluation of Bupropion and	Bupropion<br><br>Citalopram		October 2012-March 2013	1	- ICARS.<br>- FARS.<br>*- Comparison of ICARS and FARS.*	- Descriptive results, no statistical results available.

	Citalopram in an individual with FRDA.	Placebo				*- Hamilton depression rating scale.*	
22	US- EU- A phase Iia clinical trial to test the safety and efficacy interferon gamma treatment in elevating frataxin levels in Friedreich Ataxia patients.	Interferon gamma	2	May 2013- July 2014	10	- Change in cellular Frataxin.<br>*- Safety and tolerability of IFNγ by means of standard clinical and haematological criteria.*	
23	**US- Open-label Pilot Study of Interferon Gamma-1b (ACTIMMUNE®) for the Treatment of Friedreich Ataxia**	Interferon gamma-1b	2	August 2013- October 2014	12	- Change in whole blood frataxin levels.<br>*- Change in FARS score.*<br>*- T25FW.*<br>*- 9HPT.*<br>*- Visual acuity tests.*	**- Significant change in frataxin levels over 12 weeks.**<br>**- Changes in FARS score over 12 weeks equivalent to 18 months disease progression.**<br>- No significant correlation between changes in frataxin levels and changes in FARS score.<br><br>***Published results (Seyer et al., 2015)***
23	**US- Randomized, multicenter, double-blind, placebo-controlled, efficacy, safety, and pharmacokinetic study of ACTIMMUNE® (Interferon γ-1b) in children and young adults with Friedreich Ataxia**	Interferon gamma-1b<br><br>Placebo	3	June 2015- November 2016	92	- Change from baseline to week 26 in mFARS neuro score.<br>*- Change from baseline to week 26 in ADL score.*<br>*- Change from baseline at week 26 in T25FW.*<br>*- Number of mFARS neuro responders and non-responders at week 26.*<br>*- Change from baseline to week 26 in total FARS score.*	***Published results (Lynch, Hauser, et al., 2019)*** *(see next entry)*
23	**US- Multicenter, safety and efficacy, open-label extension study of ACTIMMUNE® (Interferon γ-1b) in children and young adults with Friedreich Ataxia**	Interferon gamma-1b	3	December 2015-March 2017	86	- Number of participants with TEAEs, SAEs, and discontinuations due to Aes.<br>- Number of participants with positive/negative neutralizing antibody (Nab) and anti-drug antibody (ADA) tests.<br>*- Change from baseline to week 26 in the mFARS neuro score.*<br>*- Change from baseline to week 26 in ADL score.*<br>*- Change from baseline at week 26 in T25FW.*<br>*- Number of FARS-m neuro responders and non-responders at week 26.*<br>*- Change from baseline to week 26 in total FARS score.*	- No effects on frataxin levels.<br>- No effects on mFARS or the other neurological measures.<br><br>***Published results (Lynch, Hauser, et al., 2019)***
23	US- Long-Term Safety Extension Study of ACTIMMUNE® (Interferon γ-1b) in children and young adults with Friedreich Ataxia	Interferon gamma-1b	3	June 2016- March 2017	38	- Number of participants with treatment-emergent adverse events (Aes), and discontinuations due to Aes.	

24	US- An open-label study of the effects of Acetyl-L-Carnitine on cardiovascular outcomes in Friedreich Ataxia.	Acetyl-L-Carnitine	--	August 2013-June 2017	20	- Changes in cardiac functioning in patients with FRDA between study endpoint and baseline.<br>- Changes in FRDA symptoms and severity (as measured by clinical rating scales) compared to baseline.<br>*- Changes in patient global impression of improvement.*<br>*- Changes in T25FW.*<br>*- Changes in frequency and severity of Aes.*<br>*- Changes in SF-36.*<br>*- Changes in clinical global impression of improvement.*	- No posted or published results found.
25	US- A Phase 1, randomized, double-blind, placebo-controlled, multicenter, single and multiple ascending dose study to evaluate the safety, tolerability, pharmacokinetics, and pharmacodynamics of oral VP 20629 in adult subjects with FRDA.	VP 20629<br><br>Placebo	1	August 2013-June 2015	46	- Number of participants with TEAEs and TESAEs.<br>- Number of participants with clinically significant laboratory abnormalities recorded as TEAEs.<br>- Number of participants with vital signs abnormalities recorded as TEAEs.<br>- Number of participants with clinically relevant EKG abnormalities recorded as Aes.<br>*- Maximum observed serum concentration of VP 20620 and its metabolite (VP 20631) for single dose and multiple dose groups.*<br>*- Time of maximum observed plasma concentration of VP 20620 and its metabolite (VP 20631) for single dose and multiple dose groups.*<br>*- Area under the plasma concentration versus time curve (AUC) of VP 20620 and its metabolite (VP 20631) for single dose and multiple dose groups.*<br>*- Area under the plasma concentration versus time curve (AUC[0-8]) of VP 20620 and its metabolite (VP 20631) for single dose and multiple dose groups.*<br>*- Area under the plasma concentration versus time curve to the last measurable plasma concentration (AUCt) of VP 20620 and its metabolite (VP 20631) for single dose and multiple dose groups.*<br>*- Terminal plasma half-life (t1/2) of VP 20620 and its metabolite (VP 20631) for single dose and multiple dose groups.*<br>*- Volume distribution of VP 20620 for single dose and multiple dose groups.*<br>*- Total body drug clearance of VP 20620 for single dose and multiple dose groups.*<br>*- Elimination rate constant of VP 20620 and its metabolite (VP 20631) for single dose and multiple dose groups.*<br>*- Cumulative amount excreted into the urine for unchanged VP 20620 and its metabolite (VP 20631) for single dose and multiple dose groups.*<br>*- Percentage of drug excreted in urine of VP 20620 for single dose and multiple dose groups.*	

						- Renal clearance of VP 20620 for single dose and multiple dose groups.	
26	EU- Pilot study of Incretin analogues as new therapeutics for Friedreich Ataxia.	Exenatide<br><br>Liraglutide	4	October 2014 -	70	- Change in frataxin expression measured in PBMCs, platelets and buccal swabs.<br>- SARA.<br>- INAS.<br>- ADL.	**- Exenatide treatment successfully induces frataxin expression in patients' platelets.**<br>- No effects of Exenatide on SARA, INAs, or ADL after 5-week treatment.<br><br>***Published results (Igoillo-Esteve et al., 2020)***
27	**US- EU- A Phase 2 Study of the safety, efficacy, and pharmacodynamics of RTA 408 in the treatment of Friedreich Ataxia (MOXIe)**	Omaveloxolone<br><br>Placebo	2	January 2015- December 2022	172	- Change in the mFARS.<br>- Change of peak workload (watts/Kg) during exercise testing.	**- Significant improvement in mFARS score at 48 weeks.**<br>**- Significant improvement in FA-ADL score.**<br>- No effects on 9HPT or T25FW.<br>- No effects on LCLA.<br>- No effects on peak workload.<br><br>***Published results (Lynch et al., 2021; Lynch, Farmer, et al., 2019)***
28	**US- Open-label pilot study of Methylprednisolone for the treatment of patients with Friedreich Ataxia**	Methylprednisol one	1	June 2015- April 2018	11	- Change in the T25FW.<br>*- Change in the FARS score.*<br>*- Change in the 1MWD.*<br>*- Change in in the 9HPT.*	- No significant effects in the T25FW.<br>*- No significant effects in the FARS score.*<br>*- No significant effects in the 9HPT.*<br>***- Significant effects in the 1MWD only in the paediatric cohort.***<br><br>**- Published results (Patel et al., 2019)**
29	**US- A randomized, double-blind, controlled study to assess the safety, tolerability, and pharmacokinetics of RT001 in patients with Friedreich Ataxia.**	RT001	1-2	August 2015- June 2016	19	- Number of patients with Aes.<br>*- Pharmacokinetics: area under the concentration-time curve after a single dose.*<br>*- Pharmacokinetics: maximum observed plasma concentration after a single dose.*<br>*- Pharmacokinetics: time to reach maximum plasma concentration after a single dose.*<br>*- Pharmacokinetics: maximum observed plasma concentration after final dose on day 28.*<br>*- Pharmacokinetics: terminal half-life estimation after final dose on day 28.*<br>*- Change from baseline at 28 days in the T25FW.*<br>*- Change from baseline at 28 days in the FARS.*<br>*- Change from baseline at 28 days in peak workload for the treated population vs. the comparator population.*	*- No significant effects on T25FW.*<br>*- No significant effects on FARS.*<br>***- Significant improvement in peak workload.***<br><br>***Published results (T. Zesiewicz, Heerinckx, et al., 2018)***
30	US- Open-label biomarker study of Rosuvastatin (Crestor) for the treatment of patients with Friedreich Ataxia	Rosuvastatin	1	May 2016- August 2017	12	- Change in ApoA-1 serum protein levels from baseline to Week 12 visit.<br>*- Serum ApoA-1 protein levels collected at baseline and again at the Week 12 visit.*<br>*- Change in frataxin levels from baseline to Week 12 visit.*<br>*- Change in platelet metabolism from baseline to Week 12 visit.*	
31	**US- EU- Safety and efficacy of γ-IFN in Friedreich Ataxia**	Interferon gamma	2	June 2016- December 2017	12	- Number and severity of adverse drug reactions.<br>*- Changes in SARA score.*<br>*- Change in BOLD signal during finger tapping.*	- The treatment was safe, with only 2 serious adverse effects.<br>***- Significant effects on SARA scores: scores increased in the 6-12 months prior to medication start, did not***

						- Changes in RNFL (retinal nerve fibre layer) thickness.<br>*- Thickness of ventricular wall as measured by EcoCG.*<br>*- Frataxin levels in cell lysates prepared from peripheral blood mononuclear cells.*<br>*- Changes in quality-of-life measure (SF36).*<br>*- Changes in fractional anisotropy (FA) in the cerebellar white matter, the long and the commissural tracts.*<br>*- Changes in measure of disability (WHO-DAS 2.0)*<br>*- Changes in Mean diffusivity (MD) in the cerebellar white matter, the long and the commissural tracts.*	***significantly change during the treatment, and resumed a moderate uptrend after the discontinuation of the treatment.***<br>***- The sensorimotor network, default mode network, and left fronto-parietal network showed a significantly modified activity between the start of the treatment and 6 months after the end of the treatment***<br>***- Significant increased activation of the left primary motor cortex between 6 months prior to the start of the treatment and 6 months after the start during the movement of the dominant hand. These changes negatively correlate with SARA scores changes.***<br>*- No changes in RNFL thickness.*<br>***- Significant effects on interventricular septal wall thickness: reduced during treatment and followed by a rebound 6 months after termination of treatment.***<br>***- Significant reduction of FA in the white matter of the left superior cerebellar peduncle 6 months after the end of the treatment compared with the start of the treatment. No significant changes were observed along the intermediate time points.***<br>*- No changes in MD.*<br>***- Significant correlation between fMRI changes and SARA scores.***<br><br>***Published results (Vavla, Arrigoni, et al., 2020; Vavla, D'Angelo, et al., 2020)***
32	US- A study to characterize the cardiac phenotype of individuals with FRDA (CARFA study)	Exercise-stress test	--	July 2016-September 2018	40	- Exercise-stress test.<br>- Cardiac magnetic resonance imaging (CMR).<br>- Echocardiogram.<br>- Level of cardiac biomarkers in serum.<br>- Fatigue severity scale.	
33	**US- A Phase II, open label prospective single centre drug study evaluating the safety and efficacy of (+)- Epicatechin in subjects with Friedreich Ataxia.**	(+)- Epicatechin	2	September 2016-December 2018	10	- FARS.<br>- Change in ventricular hypertrophy as shown on cardiac MRI.	- No statistically significant improvements in mFARS scores, including 9HPT and 8-meter walk test.<br>**- Improvements in cardiac structure and function.**<br><br>***Published results (Qureshi et al., 2021)***
34	**US- Rehabilitative trial with cerebello-spinal tDCS in Neurodegenerative Ataxia**	Device: anodal cerebellar and cathodal spinal tDCS<br><br>Sham cerebellar and sham spinal tDCS		February 2017-December 2018	21	- Change in ICARS score from baseline.<br>- Change in SARA score from baseline.<br>*- Change in 9HPT from baseline.*<br>*- Change in 8MW from baseline.*<br>*- Change in cerebellar brain inhibition (CBI) measurements from baseline.*<br>*- Change in SF36 from baseline.*	**- Significant effects on ICARS score.**<br>**- Significant effects on SARA score.**<br>***- Significant effects on 9HPT.***<br>*- No effects on 8MW.*<br>***- Significant effects on CBI.***<br>*- No effects on SF36.*<br><br>***Published results (Benussi et al., 2018)*** *Only 1 participant with FRDA.
35	**US- A Phase 2, randomized, double-blind, placebo-**	TAK-831<br><br>Placebo	2	November 2017-	67	- Change from baseline in the inverse time to complete the 9HPT.	- No significant effects on any index.<br><br>***Published results (Wang et al., 2021)***

	**controlled, parallel-arm study to evaluate efficacy, tolerability, and pharmacokinetics of multiple doses of oral TAK-831 in adult subjects with Friedreich Ataxia.**			December 2018		*- Change from baseline in the ADL component score of the FARS.*<br>*- Change from baseline in the inverse time to complete the 9HPT.*<br>*- Change from baseline in the ADL component individual item scores.*<br>*- Change from baseline in the mFARS.*<br>*- change from baseline in the mFARS-neuro subscales scores.*<br>*- Change from baseline in the mFARS-neuro individual item scores.*<br>*- Change from baseline in the T25FW.*<br>*- Change from baseline in the 9HPT and T25FW composite score.*<br>*- Change from baseline in LCLA test score.*<br>*- Number of participants by CGI-I (Global Change) score categories.*<br>*- Number of participants by PGI-I (Global Change) score categories.*<br>*- Number of participants by CGI-I (Upper Extremity Functional Change) score categories.*<br>*- Number of participants by PGI-I (Upper Extremity Functional Change) score categories.*<br>*- Number of participants by CGI-S categories relative to baseline.*<br>*- Number of participants by PGI-S categories relative to baseline.*<br>*- Number of participants by CGI-S (Upper Extremity Functional Severity) score categories relative to baseline.*<br>*- Number of participants by PGI-S (Upper Extremity Functional Severity) score categories relative to baseline.*<br>*- Change from baseline in the ADL component score for upper limb function items of the FARS.*<br>*- Number of participants with at least 15% or at least 20% reduction in 9HPT completion time from baseline.*
36	EU- The pharmacological effects of granulocyte-colony stimulating factor (GCSF) on Frataxin expression in patients with Friedreich Ataxia.			January 2018-December 2019	7	- Efficacy criteria: analysis of Frataxin protein and gene expression in peripheral blood mononuclear cells, analysis of related anti-oxidant enzymes in peripheral blood mononuclear cells, Frataxin protein and gene expression in platelets.<br>- Safety criteria: clinical history and examination; hematological and biochemical screening before, during and after therapy.
37	US- EU- A double-blind, placebo-controlled study on the effects of MIN-102 on biochemical, imaging, neurophysiological, and	MIN-102<br><br>Placebo	2	March 2019-September 2020	39	- Change from baseline in spinal cord area cervical segment C2-C3.

	clinical markers in patients with FRDA						
38	US- A randomized, double-blind, controlled, Phase 2/3 study to assess efficacy, long term safety and tolerability, of RT001 in subjects with FRDA.	RT001<br><br>Placebo	3	October 2019-August 2021	65	- Changes from baseline to 11 months in maximum consumption of oxygen using CPET.<br>*- Change from baseline in the timed 1MWD.*	- No significant effects of the therapy on maximum consumption of oxygen.<br>- No significant effects on timed 1MWD.
39	US- A Phase 1 single ascending dose study to assess the safety, tolerability, pharmacokinetics, and pharmacodynamics of subcutaneous CTI-1601 versus placebo in subjects with Friedreich Ataxia.	CT-1601<br><br>Placebo	1	December 2019-October 2020	28	- Number of participants with TEAEs.<br>- Number of TEAEs by System Organ Classification.<br>*- Pharmacokinetics: Area under the concentration-time curve after a single dose.*<br>*- Pharmacokinetics: Maximum observed plasma concentration after a single dose.*<br>*- Pharmacokinetics: time to reach maximum plasma concentration after a single dose.*<br>*- Pharmacokinetics: Area under the concentration-time curve from time 0 to infinity.*<br>*- Pharmacokinetics: Area under the concentration-time curve from time 0 through the last measurable point.*<br>*- Pharmacokinetics: apparent total plasma clearance.*<br>*- Pharmacokinetics: Terminal half-life estimation.*<br>*- Pharmacokinetics: apparent volume of distribution.*<br>*- Changes from baseline in Frataxin levels in buccal cell.*<br>*- Changes from baseline in Frataxin levels in whole blood.*<br>*- Changes from baseline in gene expression profiling.*	
39	US- A Phase 1 multiple ascending dose study to assess the safety, tolerability, pharmacokinetics, and pharmacodynamics of subcutaneous CTI-1601 versus placebo in subjects with Friedreich Ataxia	CTI-1601<br><br>Placebo	1	July 2020 – March 2021	27	- Number of participants with TEAEs.<br>- Number of participants with TEAEs by System Organ Classification.<br>*- Pharmacokinetics: Maximum concentration after multiple doses.*<br>*- Pharmacokinetics: Minimum or trough plasma concentration after multiple doses.*<br>*- Pharmacokinetics: Area under the curve from time 0 through the last measurable point.*<br>*- Pharmacokinetics: Terminal half-life estimation.*<br>*- Changes from baseline in Frataxin levels in buccal cell.*<br>*- Changes from baseline in levels of protein markers in buccal cell.*<br>*- Changes from baseline in gene expression in buccal cell.*<br>*- Changes from baseline in Frataxin levels in platelets.*<br>*- Changes from baseline in gene expression in whole blood.*	

						- Changes from baseline in Frataxin levels in skin punch cells.<br>*- Changes from baseline in levels of defined protein markers in blood.*<br>*- Changes from baseline in levels of specialized lipids in blood.*
39	US- A Phase 2, randomized, double-blind, placebo-controlled, dose exploration study to assess the safety, pharmacokinetics, and pharmacodynamics of subcutaneous CTI-1601 in adult subjects with Friedreich Ataxia.	CTI-1601<br><br>Placebo	2	September 2022-December 2023	15	- Number of participants with TEAEs.<br>- Maximum observed plasma concentration of CTI-1601 after multiple doses.<br>- Area under the concentration time curve of CTI-1601 from time 0 through the last measurable time point.<br>- Time to maximum observed plasma concentration of CTI-1601 after multiple doses.<br>- Time to last observed plasma concentration of CTI-1601 after multiple doses.<br>- Changes from baseline in frataxin levels in buccal cells.<br>- Changes from baseline in frataxin levels in skin punch cells.
39	US- An open label extension study of CTI-1601 in subjects with Friedreich's ataxia (Jive)	CTI-1601<br><br>Placebo	2	January 2024-January 2027		- Number of participants with TEAEs.<br>- Number of subjects with abnormal laboratory test results.<br>- Change from baseline in ECG parameters.<br>- Change from baseline in left ventricular ejection fraction.<br>- Change from baseline in left ventricular end-diastolic volume.<br>- Change from baseline in blood pressure.<br>- Change from baseline in pulse.<br>- Change from baseline in temperature.<br>- Change from baseline in respiration rate.<br>- number of subjects with any suicidal ideation or behaviour of the C-SSSR.<br>- Change from baseline at each collection timepoint in tissue frataxin concentrations.<br>- Change from baseline in 9-HPT.<br>- Change from baseline in T25-FW.<br>- Change from baseline in mFARS.<br>- Change from baseline in ADLs. (FARS-ADL).<br>- Change from baseline in MFIS.<br>- Change from baseline in Functional Staging for Ataxia.<br>- PGI-C Scale.<br>- CGI-C Scale.<br>- Mean maximum observed concentration.<br>- Mean time of maximum observed concentration.<br>- Area under the concentration time curve of from time 0 through the last quantifiable concentration.<br>- Area under the concentration time curve for the dosing interval.

						- Concentration reached immediately before the next dose is administered.	
40	US- NAD+ precursor supplementation with exercise training to increase aerobic capacity in Friedreich Ataxia.	Nicotinamide Riboside<br><br>Placebo<br><br>Exercise intervention	--	September 2020- December 2025	72	- Within participant change in VO2 maximal measured by completion of the Exercise Stress Test (EST).<br>*- Within-participant change in whole body insulin sensitivity assessed by a stable isotope tracer-enhanced oral glucose tolerance test (OGTT).*	
40	US- A Phase 2a study of NAD+ precursor supplementation in Friedreich Ataxia	MIB-626 (nicotinamide adenine dinucleotide precursor)	2	May 2021- September 2023	10	- Incidence of treatment-emergent adverse events as assessed by Common Terminology Criteria for Adverse Events version 5.0.<br>*- Cardiac 31-Phosphorus-MRS: phosphocreatine/ATP Ratio.*<br>*- Post-exercise CrCEST.*<br>*- Grip Strength.*<br>*- Concentration of NAD+ in Whole Blood.*	
41	US- EU- A Phase 2 clinical trial to test the safety and efficacy of Etravirine in Friedreich Ataxia patients.	Etravirine	2	September 2020- November 2022	30	- Adverse event number and severity.<br>*- VO2 max increase.*<br>*- SARA score.*<br>*- Cardiac wall thickness.*<br>*- Frataxin measurement.*<br>*- SF36 for quality of life.*	- No posted or published results found.
42	US- Home based tele-exercise for people with chronic neurological impairments	Seated exercise	--	January 2021- December 2023	60	- Change in heart rate.	
43	US- Vasculopathy and remodeling of coronary arteries in Friedreich Ataxia patients.	--	--	February 2021- August 2023	6	- Vasomotor tone.	
44	US- Evaluation of the effect of Artesunate in Friedreich Ataxia (FA) Phase I-II efficacy-toxicity of Artesunate in Friedreich Ataxia.<br><br>EU- Phase I-II efficacy-toxicity of artesunate in Friedreich Ataxia	Artesunate	1-2	May 2022- April 2024	20	- Search for the maximal tolerated and effective dose of oral artesunate to regulate iron homeostasis and Transferrin 1 receptor immunofluorescence in peripheral blood mononuclear cells.<br>*- Incidence of adverse events with Artesunate in FRDA patients.*<br>*- Type of adverse events with Artesunate in FRDA patients.*<br>*- Impact of stopping an effective dose of Artesunate on the regulation of iron homeostasis and TfR1 immunofluorescence.*	
45	US- Pilot trial about the effects of Calcitriol's treatment in the neurological function and Frataxin's level in Friedreich Ataxia patients.	Calcitriol	4	August 2021- September 2022	20	- Changes in the SARA scale.<br>- Changes in the 9HPT.<br>- Changes in the F8WT test.<br>- Changes in the PATA rate test.<br>- Changes in Barthel Index for Activities of Daily Living.<br>- Changes in SF36 questionnaire.<br>- Changes in Frataxin levels.	- No results involving neurological evaluation posted or published.<br>- On the effect of Calcitriol increasing frataxin levels in FRDA, see (Britti et al., 2021)

						- *Assessing side effects with a hypercalcemia symptoms questionnaire.*<br>- *Assessing hypercalcemia risk with an EKG.*<br>- *Assessing hypercalcemia risk with a blood test.*	
46	US- Phase Ia Study of AAVrh.10hFXN gene therapy for the cardiomyopathy of Friedreich Ataxia.	Prednisone	1	February 2022- December 2029	10	- Safety of AAVrh.10hFXN.<br>- *Change in cardiopulmonary exercise testing.*<br>- *Change in cardiac-relevant parameters in cardiac-magnetic resonance scans.*<br>- *Change in cardiac-relevant parameters in echocardiograms.*<br>- *Change in arrhythmias with 24-hour monitoring.*	
47	US- A pilot investigator-initiated study to evaluate the safety, tolerability and efficacy of Elamipretide in the treatment of advanced symptoms of Friedreich Ataxia.	Elamipretide	1-2	March 2022- June 2024	18	- Change in high contrast visual acuity: ETDRS high contrast visual acuity chart.<br>- *Change in low contrast visual acuity: ETDRS low contrast visual acuity chart.*<br>- *Change in retinal nerve fiber layer by Optical Coherence Tomography (OCT).*<br>- *Change in visual quality of life by Visual Functioning Questionnaire (VFQ).*<br>- *Change in cardiac strain.*<br>- *Change in cardiac fibrosis.*<br>- *Change in cardiac stroke volume.*	
48	US- A Phase 1a, randomized, double-blind, placebo-controlled, single ascending dose study of DT-216 in adult patients with Friedreich Ataxia	DT-216	1	March 2022- December 2022	39	- Frequency of TEAEs.<br>- *Maximum plasma concentration of DT-216.*<br>- *Time to maximum plasma concentration of DT-216.*<br>- *Area under the concentration-time curve (AUD).*<br>- *Frataxin expression.*	
48	US- A Phase 1b, randomized, double-blind, placebo-controlled, multiple ascending dose and multi-dose study of DT-216 in adult patients with Friedreich Ataxia.	DT-216<br><br>Placebo	1	September 2022- September 2023	38	- Frequency of TEAEs.<br>- Maximum plasma concentration of DT-216.<br>- Time to maximum plasma concentration of DT-216.<br>- Area under the concentration-time curve of DT-216.<br>- Frataxin expression.	
49	US- EU- A randomized, double-blind, placebo-controlled, parallel-group, multicentre study of the efficacy and safety of Nicotinamide in patients with Friedreich Ataxia	Nicotinamide<br><br>Placebo	2-3	April 2023- December 2025	225	- SARA score.<br>- *Progression of quality-of-life measures via questionnaire EuroQol Group (1990).*<br>- *mFARS.*<br>- *Progression of cerebellar severity measured by CCFS score.*<br>- *CGI-C.*<br>- *Safety issues measured by appearance of Aes/SAEs.*	- No posted or published results found.
50	US- A Phase 1/2 study of the safety and efficacy of LX2006 gene therapy in participants with cardiomyopathy	LX2006	1-2	August 2022- September 2029	10	- TEAEs and TESAEs.<br>- *Change from baseline in LVMi.*<br>- *Change from baseline in LVEF.*<br>- *Change from baseline in cardiac fibrosis as measured by cardiac MRI.*	

	associated with Friedreich Ataxia.					*- Change from baseline in measures of cardiopulmonary exercise tolerance.*<br>*- Presence of severity of cardiac arrythmias.*	
51	US- EU- A randomized, parallel-arm, double-blind, placebo-controlled study with open-label extension to assess the efficacy and safety of Vatiquinone for the treatment of Friedreich Ataxia (MOVE-FA)	Vatiquinone<br><br>Placebo	2-3	December 2020- September 2023	146	- Change from baseline in mFARS.<br>*- Change from baseline in FARS-ADL.*<br>*- Change from baseline in 1MWD.*<br>*- Number of falls through week 72.*	- No posted or published results found.
51	US- EU- An open-label study to evaluate pharmacokinetics, safety, and efficacy of Vatiquinone in children with Friedreich Ataxia younger than 7 years of age.	Vatiquinone	2	August 2022- August 2024	5	- Plasma concentration of Vatiquinone.<br>- Area under the curve (AUC) of Vatiquinone.<br>- Number of participants with adverse effects.	
51	US- Long-term open-label study to assess the safety and efficacy of Vatiquinone in patients with Friedreich Ataxia	Vatiquinone	3	November 2022- December 2027	140	- Number of participants with adverse events.<br>*- Change from baseline in the mFARS at year 5.*	- No posted or published results found.
52	US-Respiratory training in Friedreich;s Ataxia	--	N/A	August 2024- March 2025	8	- Force vital capacity.<br>- Maximal inspiratory pressure (MIP)<br>- Maximal expiratory pressure (MEP).<br>- Sniff nasal inspiratory pressure (SNIP).<br>- Swallowing.<br>- Surface electromyography (sEMG)<br>- Diaphragm ultrasound.	- No posted or published results found.
53	US-A Study of Omaveloxolone in children with Friedreich's ataxia (BOLD)	Omaveloxolone	1	July 2024- February 2030		- Apparent clearance of Omaveloxolone.<br>- Maximum concentration of Omaveloxolone.<br>- Volume of distribution of amaveloxolone.<br>- Area under the plasma concentration of omaveloxolone.<br>- Area under the plasma concentration-time curve from 0 to tlast of Omaveloxolone.<br>- etc.<br>-	
54	US-A study of ASP2016 in adults who have heart disease associated with Friedreich Ataxia	ASP2016	1	September 2024-January 2031	14	- Number of participants with adverse events.<br>- A series of cardiac indicators.	
