# Supplementary material for: Clinical and cognitive assessment in Friedreich ataxia clinical trials: a review
Source: Front Neurol. 2025 May 22;16:1558493. doi: 10.3389/fneur.2025.1558493 (PMC12142069; doi:10.3389/fneur.2025.1558493)
Supplement: Supplementary file 3 [file Data_Sheet_3.PDF]

Table 3. Summary of publications found in the PubMed database search. Studies included in the table (1) use some kind of neurological evaluation, (2) include mainly FRDA patients, and (2), are not included in Table 3. In bold, significant results. **6MWT:** Six-minute walk test; **9HPT:** 9-Hole Peg Test; **ADL:** Activities of Daily Living; **ATP:** Adenosine Triphosphate; **EKG**: Electrocardiography; **EMG:** Electromyography; **FARS**: Friedreich Ataxia Rating Scale; **FASS:** Functional Ataxia Scoring Scale; **ICARS**: International Cooperative Ataxia Rating Scale; **MRI:** Magnetic Resonance Imaging; **MT:** Movement Time; **N:** Number of participants; **RT:** Reaction Time; **SARA:** Scale for the Assessment and Rating of Ataxia; **SF-36:** 36-item short form survey for quality of life.

**Authors**	**N**	**Description**	**Findings**
(Peterson et al., 1988)	16	Treatment: Amantadine Hydrochloride<br>Evaluation:<br>- FASS, modified.	**- Total disability score significantly improves after a single dose treatment. Effects gradually dissipates over 4 hours.**
(Botez et al., 1991)	17	Treatment: Amantadine Hydrochloride<br>Evaluation:<br>- RT, MT.	- Improvement only in two out of four MT measures with no improvement in RT.
(Filla et al., 1993)	14	Treatment: Amantadine Hydrochloride<br>Evaluation:<br>- FASS, modified.	- No significant effects on FASS.
(Wessel et al., 1995)	19	Treatment: Laevorotatory form of hydroxytryptophan<br>Evaluation:<br>- Clinical score.<br>- Posturography.<br>- Measurement of grip force.<br>- Rapid-syllable repetition rate.	- No significant effects.
(Trouillas et al., 1995)	26	Treatment: laevorotatory form of 5-Hydroxytrytophan.<br>Evaluation:<br>- Ataxia rating scale including five subtests of a kinetic score and eight subtest of a static score (each within a 0-4 range).<br>Kinetic score:<br>• Quality of finger-nose test, R<br>• Quality of finger-nose test, L<br>• Quality of knee-tibia test, R<br>• Quality of knee-tibia test, L<br>• Quality of drawing Archimedes' spiral<br>Static score:<br>• Quality of standing in a natural position<br>• Body oscillations in natural position<br>• Leg muscle movements in natural position<br>• Quality of standing with feet together.<br>• Leg muscle movements with feet together<br>• Quality of gait<br>• Quality of sitting position<br><br>- Time to pronounce a standard sentence.<br>- Time to write name.	**- Only significant effects on the kinetic scores of the ataxia rating scale.**
(Botez et al., 1996)	27	Treatment: amantadine hydrochloride.<br>Evaluation:<br>- Simple visual and auditory RT and MT.	- No significant effects on RT.<br>**- Significant effects on visual MT with the left hand and auditory MT with the left hand.**
(Sorbi et al., 2000)	24	Treatment: L-acetylcarnitine.<br>Evaluation:<br>- Clinical rating score	**- Mild improvement in clinical scores of coordination after 3 and 6 months of treatment.**
(Schöls et al., 2001)	9	Treatment: Idebenone.<br>Evaluation:<br>- P magnetic resonance spectroscopy.<br>- ICARS.<br>- Plugging subtest of the motor performance test according to Schoppe.<br>- Electrocardiogram.	- No effects observed in P magnetic resonance spectroscopy.<br>- No significant effects on ICARS.<br>- No significant effects on motor performance test.<br>- No changes in electrocardiogram.
(Lodi et al., 2001)	10	Treatment: Coenzyme Q10, Vitamin E.<br>Evaluation:<br>- ICARS.<br>- Echocardiographic measurements.<br>- Measurements of cellular bioenergetics.	- No significant effects on ICARS scores.<br>**- Significant increase in the cardiac phosphocreatine to ATP ratio and on the maximum rate of skeletal muscle**

			**mitochondrial ATP production (cellular bioenergetics).**
(Artuch et al., 2002)	9	Treatment: Idebenone.<br>Evaluation:<br>- ICARS.<br>- EMG.<br>- Never conduction velocity (NCV)<br>- Somatosensory and visual potentials.	**- Significantly negative correlations between serum Idebenone and ICARS score after 12 months of treatment.**<br>**- Significantly positive correlations between Idebenone values and the percentage of difference between the ICARS scores before and 12 months after the start of the therapy.**<br>**- Significant reduction comparing the ICARS scores in baseline conditions and after 3, 6, and 12 months of treatment.**<br>- No differences in echocardiographic and neurophysiological measurements.
(Buyse et al., 2003)	8	Treatment: Idebenone.<br>Evaluation:<br>- Several measures of cardiac structure and function.<br>- Biochemical markers.<br>- Cooperative Ataxia Group (CAG) rating scale for drug trial for FRDA.	**- Significant improvement of cardiac function.**<br>- No significant protective effects on CAG scores: progressive increase in total AG scores over time.<br>- Changes in biochemical markers do not correlate with cardiac improvement.
(Mariotti et al., 2003)	28	Treatment: Idebenone.<br>Evaluation:<br>- ICARS.<br>- Echocardiographic measurements.	- No significant changes in ICARS scores.<br>**- Significant improvement in echocardiographic measurements.**
(Schöls et al., 2005)	16	Treatment: L-carnitine.<br>Evaluation:<br>- Mitochondrial ATP production measured as phosphocreatine.<br>- ICARS.<br>- Cardiac hypertrophy.	- No significant effects on phosphocreatine recovery compared to placebo.<br>- No significant changes in ICARS.<br>- No significant changes in cardiac hypertrophy.
(Hart et al., 2005)	10	Treatment: coenzyme Q10 and Vitamin E.<br>Evaluation:<br>- Vitamin E and CoQ10 levels in serum.<br>- ICARS.<br>- Number of thumb hits on a hand tally.<br>- Echocardiography.<br>- Magnetic resonance spectroscopy	**- Prolonged improvement in cardiac and skeletal muscle bioenergetics.**<br>**- Total ICARS scores did not worsen over the 47-month trial period.**
(Ribaï et al., 2007)	88	Treatment: Idebenone<br>Evaluation:<br>- ICARS.<br>- Quantitative writing test.<br>- Echocardiography, EKG, Holter monitoring.<br>- Electrooculography.	- ICARS worsened regardless of the Idebenone treatment.<br>- ICARS worsened faster in patients with early onset (<15 years age)<br>- ICARS scores reach ceiling values after long disease durations, preventing accurate evaluation of disease progression.
(Pineda et al., 2008)	24	Treatment: Idebenone.<br>Evaluation:<br>- ICARS.<br>- Echocardiographic measurements.	- No significant effects on echocardiographic measurements.<br>- No significant changes in ICARS scores in paediatric patients after 5 years follow-up.<br>**- Significant increase in ICARS scores in adult patients during 3 years of therapy.**
(Cooper et al., 2008)	50	Treatment: coenzyme Q10 and vitamin E.<br>Evaluation:<br>- Serum Q10 and Vitamin E.<br>- ICARS.	**- Relative to baseline, significant effect on CoQ10 and Vitamin E levels in the high- and low-dose groups.**<br>**- Compared to cross-sectional data, 49% of patients showed slower than predicted deterioration of ICARS score.**
(Velasco-Sánchez et al., 2011)	20	Treatment: Deferiprone and Idebenone.<br>Evaluation:<br>- ICARS.<br>- Echocardiographic measurements.<br>- MRI to assess brain iron deposits in the dentate nucleus.	- No significant effects on total ICARS scores.<br>**- Increase in posture and gait scores.**<br>**- Improvement of kinetic function.**<br>**- Significant reduction of iron deposits in the dentate nucleus.**
(Rufini et al., 2011)	5	Treatment: IGF-1<br>Evaluation:<br>- SARA.<br>- Echocardiogram.	- Slowed annual worsening rate of the SARA treated patients compared to controls.
(Nachbauer, Wanschitz, et al., 2011)	7	Treatment: recombinant human erythropoietin.<br>Evaluation:<br>- Relationship between frataxin levels measured in peripheral blood mononuclear cells (PBMC) and those in skeletal muscle.	**- Frataxin content is correlated in peripheral blood mononuclear cells and skeletal muscle in drug-naïve FRDA patients.**

		- SARA.	**- The aforementioned correlations remain significant after 8 weeks of treatment.**<br>**- SARA scores correlate inversely with frataxin in skeletal and PBMCs before the treatment and after 8 weeks of treatment.**<br>- Absolute changes in frataxin levels and SARA scores do not correlate.
(Mariotti et al., 2012)	16	Treatment: erythropoietin.<br>Evaluation:<br>- Frataxin levels.<br>- EPO levels.<br>- SARA.<br>- 9HPT.<br>- SF-36	- No effects on Frataxin levels.<br>- No effects on EPO levels.<br>- No effects on SARA.<br>- No effects on 9HPT.<br>- No effects on SF-36.
(Arpa et al., 2013)	9	Treatment: Darbepoetin alfa, Idebenone, and Riboflavin.<br>Evaluation:<br>- SARA.<br>- Changes in septal wall thickness and posterior wall thickness.<br>- SF-36	- No significant effects on SARA scores.<br>- No significant effects on the other evaluation parameters.
(Arpa et al., 2014)	13	Treatment: Deferiprone, Idebenone, and Riboflavin.<br>Evaluation:<br>- ICARS.<br>- ADL.<br>- Echocardiography.	- Possible slowing of annual worsening rate of the SARA score for treated patients compared to the expected rate in FRDA population.<br>- No significant effects on the other parameters.
(Sanz-Gallego et al., 2014)	5	Treatment: IGF-1<br>Evaluation:<br>- SARA.<br>- Echocardiography.	**- Significant effect on stabilising SARA scores from the first quarter of the study (4 months). Rebound effect one year after the end of the trial.**<br>- No changes in echocardiography parameters.
(Elincx-Benizri et al., 2016)	5	Treatment: Deferiprone and Idebenone.<br>Evaluation:<br>- Laboratory blood tests.<br>- SARA.<br>- FARS.<br>- SF-36.<br>- Electrocardiogram<br>- Echo-Doppler.<br>- 24-hours Holter recording.	- Authors conclude that results suggest neurological function and heart hypertrophy improvements. Data are purely descriptive. No statistical data are provided.
(Leonardi et al., 2017)	11	Treatment: wearable proprioceptive stabilizer emitting focal mechanical vibrations.<br>Evaluation at baseline (T0), after 3 weeks of device utilization (T1), after 3 weeks from device discontinuation (T2), through:<br>- SARA.<br>- 9HPT dominant hand.<br>- PATA test.<br>- 6MWT.<br>- Spatial and temporal gait parameters measured with a BTS-G-Walk inertial sensor.	**- Significant improvement in SARA, 9HPT dominant hand, PATA, 6MWT, and gait parameters between T0 and T1. No differences between T1 and T2.**
(Milne et al., 2018)	19	Treatment: Six-week rehabilitation programme.<br>Evaluation:<br>- Functional Independence Measure.<br>- Friedreich Ataxia Impact Scale.<br>- FARS.	- No significant impact on the Functional Independent Measure.<br>**- Significant differences between experimental and control groups in the Friedreich Ataxia Impact Scale body movement subscale.**<br>**- Significant within-group differences in FARS.**
